# Individual and combined associations of modifiable metabolic health and lifestyle with low back pain, neck pain, and functional limitation: evidence from a nationally cross-sectional study

**DOI:** 10.1186/s12889-026-27651-3

**Published:** 2026-05-01

**Authors:** Chengxin Xie, Qihang Li, Jingxin Xin, Xiao Jiang, Qiang He, Wenbo Bian, Dongting Yu, Yanru Lin, Zhenyu Yao

**Affiliations:** 1 Key Laboratory of Endocrine Glucose & Lipids Metabolism and Brain Aging, Ministry of Education;, Department of Endocrinology, Shandong Provincial Hospital Affiliated to Shandong First Medical University, Jinan, Shandong China; 2 Shandong Clinical Research Center of Diabetes and Metabolic Diseases, Jinan, Shandong China; 3 Shandong Institute of Endocrine and Metabolic Diseases, Jinan, Shandong China; 4 “Chuangxin China” Innovation Base of stem cell and Gene Therapy for endocrine Metabolic diseases, Jinan, Shandong China; 5 School of Clinical and Basic Medicine, Shandong First Medical University, Shandong Academy of Medical Sciences, Jinan, Shandong China

**Keywords:** Metabolic health, Lifestyle, Low back pain, Neck pain, Functional limitation, American adults

## Abstract

**Background:**

Although healthy lifestyles are prioritized for the self-management of chronic low back pain (LBP) and neck pain (NP), the individual and combined associations of metabolic health and lifestyle with LBP, NP, and related functional limitations remain unclear. This study aimed to evaluate these associations and support risk stratification across different metabolic and lifestyle profiles.

**Methods:**

This cross-sectional study used data from the US National Health and Nutrition Examination Survey (1999–2018) to analyze two cohorts: pain cohort (*n* = 10286) and functional limitation cohort (*n* = 28513). Metabolic factors included abdominal obesity, hyperglycemia, hypertension, and dyslipidemia. Lifestyle factors included smoking, alcohol consumption, diet, and physical activity. Outcomes, including LBP, NP, and back- or neck-related functional limitation (BN-FL), were assessed by self-reported questionnaires. Associations between risk factors and outcomes were assessed using multivariable logistic regression.

**Results:**

Individuals with poor metabolic-lifestyle status had the highest prevalence of LBP (42.6%), NP (21.4%), and BN-FL (17.6%). Physical inactivity showed the largest estimated population-attributable fraction (PAF) for LBP and NP (20.8% and 20.5%, respectively), while abdominal obesity (15.1%) and smoking (14.0%) showed the largest estimated PAFs for BN-FL. Poor metabolic status was associated with higher odds of LBP (OR = 1.470, 95% CI: 1.227–1.760) and BN-FL (OR = 2.404, 95% CI: 1.977–2.923), while poor lifestyle was associated with higher odds across all outcomes. Worsening lifestyle status was consistently associated with higher odds of LBP across all metabolic strata. The combination of poor metabolic-lifestyle status was associated with the highest odds of LBP (OR = 3.435, 95% CI: 2.026–5.824) and BN-FL (OR = 4.378, 95% CI: 2.347–8.167).

**Conclusions:**

Both metabolic health and lifestyles were associated with LBP and BN-FL, while only lifestyles were linked to NP. Combined unfavorable metabolic and lifestyle profiles were associated with higher odds of LBP and BN-FL, underscoring the importance of both metabolic and lifestyle modification.

**Supplementary Information:**

The online version contains supplementary material available at 10.1186/s12889-026-27651-3.

## Introduction

Low back pain (LBP) and neck pain (NP) are major public health problems worldwide, substantially reducing work productivity, quality of life, and leading to disability. According to the Global Burden of Disease Study 2021, about 619 million people suffered from LBP in 2020, and this number is projected to reach 843 million by 2050 [1]. NP is also widespread, affecting an estimated 288.7 million people globally [2]. With population ageing, urbanization, and increasingly sedentary lifestyles, this burden is expected to intensify. Despite extensive research and clinical guidelines, effective prevention and long-term management remain challenging, underscoring the urgent need for evidence-based, accessible, and nonpharmacologic approaches [3–5].

Although LBP is the leading cause of disability worldwide, it has received insufficient attention in the global health agenda [5]. The Lancet Low Back Pain Series Working Group has called for global action and proposed the positive health concept, defined as “the ability to adapt and self-manage in the face of social, physical, and emotional challenges”, as a unifying strategy to prevent long-term disability [4]. Public health programmes addressing obesity and physical inactivity could serve as models for mitigating the burden of LBP, although the independent associations between lifestyle behaviors and pain remain uncertain [5]. Given that obesity is commonly accompanied by metabolic abnormalities, targeting modifiable metabolic and behavioral risk factors offers a feasible pathway for preventing both LBP and NP and their progression to chronic disability.

However, existing evidence on these associations remains fragmented. Prior studies have reported limited or inconsistent associations between obesity, metabolic disorders, and lifestyle behaviors with LBP or NP individually [3, 6–10]. Yet, few have systematically evaluated how lifestyle factors relate to these conditions across populations with varying metabolic profiles. Beyond that, the joint associations of overall metabolic health and lifestyle patterns with LBP and NP remain poorly understood. Therefore, it is important to conduct risk stratification for conditions of high public health concern by integrating metabolic and lifestyle profiles. Such an approach can improve understanding of modifiable risk compositions, facilitate the identification of high-risk populations, and inform targeted interventions [11].

LBP and NP, although often considered together as spinal pain conditions, may differ in their underlying biological and ergonomic mechanisms. Previous literature suggests that LBP is more strongly influenced by load-bearing structures of the lumbar spine and has been linked to metabolic factors such as obesity and systemic inflammation [9, 12, 13], whereas NP is more closely associated with cervical posture, occupational or screen-based ergonomic exposures, and localized musculoskeletal strain [12, 14] These differences suggest that the relative associations of metabolic and lifestyle factors may not be uniform across these conditions, underscoring the importance of evaluating them separately.

In this study, we assessed both the individual and joint associations of metabolic and lifestyle status with LBP and NP and related functional limitations using a nationally representative dataset. We focused on four metabolic factors (abdominal obesity, hyperglycemia, hypertension, dyslipidemia) and four lifestyle factors (smoking, alcohol consumption, diet, and physical activity). Subgroup analyses by age and sex were conducted to identify population-specific patterns that could guide precision prevention strategies. In addition, we validated the results in both sedentary and non-sedentary populations.

## Materials and methods

### Data sources

Since 1999, the National Health and Nutrition Examination Survey (NHANES), has been conducted continuously in two-year cycles using a stratified, multistage probability sampling design to obtain nationally representative samples of the non-institutionalized US population. The survey protocol was approved by the Institutional Review Board of National Center for Health Statistics, and all participants provided written informed consent. Data and related documentation are publicly available at https://www.cdc.gov/nchs/nhanes/. As the data are publicly available and de-identified, additional institutional review board approval was not required.

## Study population

**Fig. 1:**
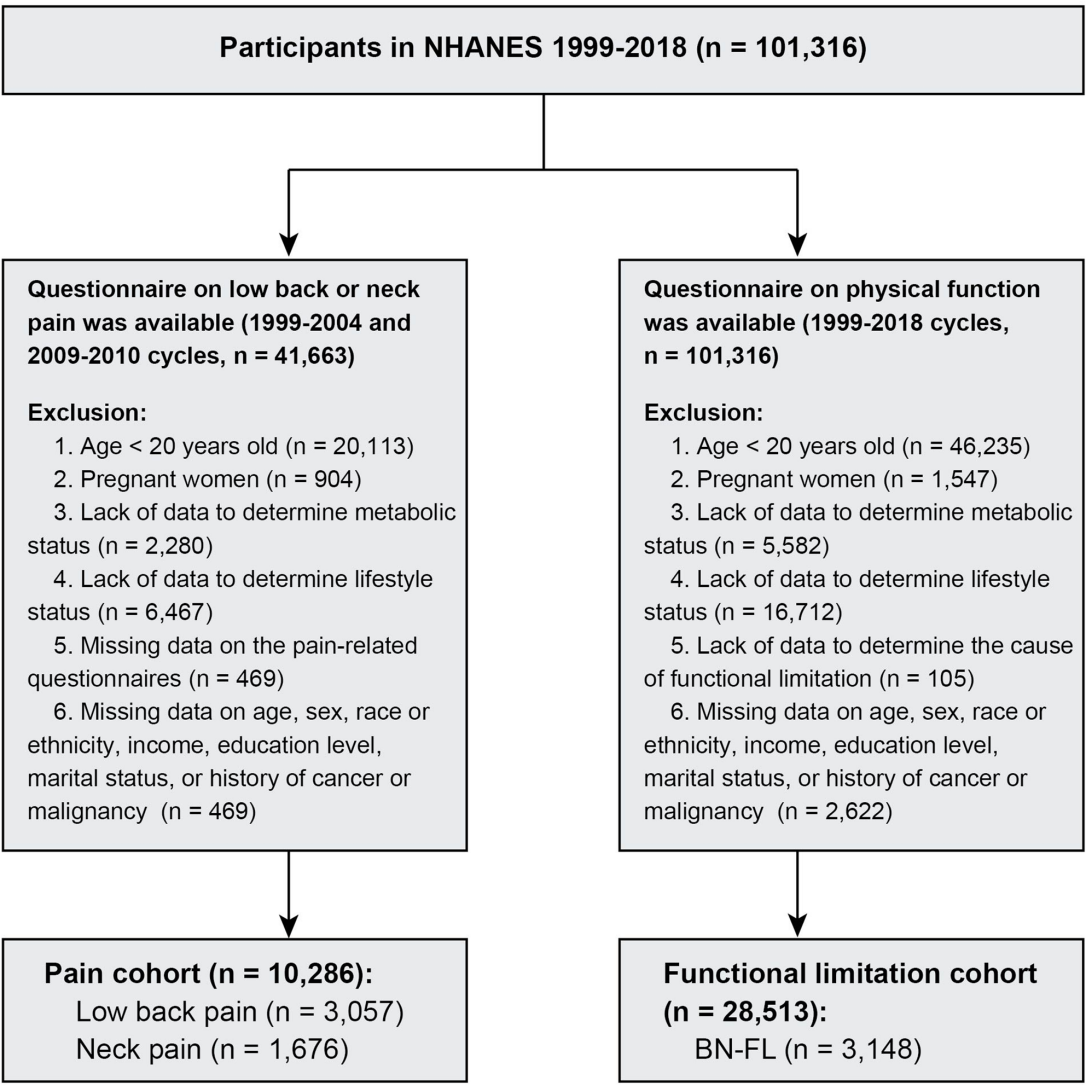
Flowchart of the participants selection. Abbreviations: NHANES, National Health and Nutrition Examination Survey; BN-FL, back- or neck-related functional limitation

Information on LBP and NP was available only in the 1999–2004 and 2009–2010 cycles; therefore, these cycles were used to construct the pain cohort. Participants were excluded if they were younger than 20 years, pregnant, or missing data on metabolic or lifestyle status, pain-related questionnaires, or covariates (age, sex, race/ethnicity, income, education, marital status, or history of cancer/malignancy). The final analytic sample for the pain cohort included 10,286 (weighted *n* = 121412851) participants (Fig. 1).

In contrast, data on functional limitation were available across all NHANES cycles from 1999 to 2018. Accordingly, a separate functional limitation cohort was constructed to maximize the use of available data and increase statistical power for this outcome. Exclusion criteria were the same as above, except that participants with missing data on the causes of functional limitation were excluded instead of those missing pain data. The final analytic sample for the functional limitation cohort included 28,513 (weighted *n* = 134005538) participants (Fig. 1). Of these, 18,302 participants had complete data on sedentary time.

## Assessment of metabolic factors

Metabolic data were obtained from anthropometric measurements, laboratory tests, and self-reported medical history. Four metabolic factors were evaluated according to the diagnostic criteria for metabolic syndrome: abdominal obesity, hyperglycemia, hypertension, and dyslipidemia [15].

Abdominal obesity was defined as a waist circumference ≥ 102 cm in men or ≥ 88 cm in women. Hyperglycemia was defined as fasting plasma glucose ≥ 6.1 mmol/L, use of hypoglycemic medication, or self-reported diabetes. Hypertension was defined as systolic blood pressure ≥ 140 mmHg and/or diastolic blood pressure ≥ 90 mmHg, self-reported hypertension, or use of antihypertensive medication. Dyslipidemia was defined as triglyceride levels ≥ 1.69 mmol/L, total cholesterol ≥ 200 mg/dL, self-reported dyslipidemia, or use of lipid-lowering medication.

Each metabolic risk factor was assigned 1 point, yielding a total metabolic score ranging from 0 to 4. This approach was chosen based on its simplicity and widespread use in epidemiological studies [11, 16, 17]. Participants were classified into three metabolic status categories: good (score = 0), moderate (score = 1–2), and poor (score = 3–4). Refer to previous studies [11, 16, 17] and based on the frequency distribution of participants (Supplementary Figure S1), this method is simple and clinically meaningful, while ensuring sufficient sample sizes for statistical analysis.

## Assessment of lifestyle factors

Lifestyle information was collected through structured questionnaires and 24-hour dietary recalls. According to the recommendations of the World Health Organization and previous studies, we assessed four lifestyle factors in the primary analysis: cigarette smoking, alcohol consumption, diet, and physical activity [17].

Current daily smoking was classified as an unhealthy level. Excess alcohol intake was defined as ≥ 2 drinks per day for women and ≥ 3 drinks per day for men [17]. Dietary quality was evaluated using the Healthy Eating Index-2020 (HEI-2020), and an unhealthy diet was defined as an HEI-2020 score within the lowest two quintiles of the distribution [17]. Physical activity was assessed based on weekly metabolic equivalent hours of leisure-time activity, and participants in the lowest two tertiles of metabolic equivalent hours were considered physically inactive [17]. Each unhealthy lifestyle factor was assigned 1 point, and participants were categorized into three groups: good (score = 0), moderate (score = 1–2), and poor (score = 3–4), similar to the classification of metabolic factors.

Daily sedentary time was assessed based on participants’ self-reported average sitting time during a typical day, including time spent at school, at home, during transportation, and during leisure activities, while excluding time spent sleeping. Participants reporting ≥ 5 h per day were classified as having sedentary behavior [18].

## Assessment of outcomes

LBP, NP, and back- or neck-related functional limitation (BN-FL) were assessed using structured questionnaires. Participants were instructed to report only pain that lasted a whole day or longer and to exclude transient or mild pain. Consistent with prior NHANES-based studies [19, 20], LBP was defined as answering “yes” to the question: “During the past 3 months, did you have low back pain?”. Similarly, NP was defined by a “yes” response to: “During the past 3 months, did you have neck pain?”. BN-FL was defined as difficulty performing any of the specified activities due to back or neck problems. Participants were asked about difficulty in performing 20 specific activities (Supplementary Table S1), and functional limitation was categorized as “some difficulty,” “much difficulty,” or being “unable to do” any activity. Those attributing their limitations to back or neck problems were classified as having BN-FL, regardless of whether they reported current LBP or NP.

### Covariates

Covariates included age, sex, race/ethnicity, income, education, marital status, and history of cancer/malignancy. Race/ethnicity were classified into four categories: non-Hispanic White, non-Hispanic Black, Mexican American, and Other. Income was assessed using the family income-to-poverty ratio. Education level was categorized as less than high school, high school or equivalent, and college or above. Marital status was categorized as married or living with a partner, never married, and widowed, divorced, or separated.

### Statistical analysis

All statistical analyses incorporated sampling weights to obtain nationally representative estimates. Baseline characteristics were compared using nonparametric Wilcoxon rank-sum tests for continuous variables and Rao-Scott adjusted chi-square tests for categorical variables. Weighted independent two-sample t tests were used to compare prevalence between groups, and weighted linear regression models were applied to assess trends across metabolic or lifestyle groups.

Associations of individual and combined metabolic and lifestyle factors with study outcomes were examined using multivariable logistic regression, and results expressed as odds ratios (OR) and 95% confidence intervals (CI). All models were adjusted for age, sex, race/ethnicity, income, education, marital status, and history of cancer/malignancy. Population attributable fraction (PAF) was estimated by simultaneously including all metabolic, lifestyle, and covariate variables in a model.

Subgroup analyses were conducted by age and sex. Age was categorized as young (20–44 years), middle-aged (45–64 years), and elderly (≥ 65 years). We conducted several sensitivity analyses. First, models were additionally adjusted for metabolic and lifestyle factors to assess their independent associations with outcomes. Second, analyses were also stratified by sedentary status to test the robustness of associations between metabolic-lifestyle status and BN-FL. Third, weighted metabolic and lifestyle scores were constructed based on regression coefficients to account for differences in the strength of associations across components.

All statistical analyses were performed using R software 4.3.3, and a *p* < 0.05 was considered statistically significant.

## Results

### Characteristics of study population

In the pain cohort, 10,286 adults were included, with a median age of 43 years. Of these, 3057 reported LBP and 1676 reported NP. Compared to participants without LBP, those with LBP had a higher proportion of abdominal obesity, hyperglycemia, hypertension, dyslipidemia, smoking, unhealthy diet habits, and physical inactivity. Participants with NP, compared to those without, had a higher proportion of dyslipidemia, smoking, unhealthy diet habits, and physical inactivity (Table 1).

**Table 1:** Characteristics of the study population

Characteristics	Pain cohort							Functional limitation cohort			
	Overall (*N* = 10286)	LBP		*P* value	NP		*P* value	Overall (*N* = 28513)	BN-FL		*P* value
		No (*N* = 7229)	Yes (*N* = 3057)		No (*N* = 8610)	Yes (*N* = 1676)			No (*N* = 25365)	Poor (*N* = 3148)	
Age, year	43 (32, 55)	43 (32, 54)	44 (33, 55)	0.061	43 (31, 55)	44 (35, 53)	0.179	44 (32, 57)	43 (31, 55)	57 (45, 67)	< 0.001
Age group, N (%)				0.326			0.032				< 0.001
20–44 years	4870 (52.7)	3510 (53.3)	1360 (51.3)		4125 (52.8)	745 (52.0)		13,250 (50.1)	12,599 (52.8)	651 (24.6)	
45–64 years	3519 (35.8)	2416 (35.3)	1103 (36.8)		2876 (35.2)	643 (38.4)		9589 (35.7)	8253 (34.8)	1336 (43.6)	
≥ 65 years	1897 (11.6)	1303 (11.4)	594 (11.8)		1609 (12.0)	288 (9.6)		5674 (14.3)	4513 (12.4)	1161 (31.8)	
Sex, N (%)				0.047			0.003				< 0.001
Female	4771 (47.8)	3280 (47.1)	1491 (49.2)		3905 (46.9)	866 (51.9)		13,265 (47.9)	11,648 (47.2)	1617 (54.8)	
Male	5515 (52.2)	3949 (52.9)	1566 (50.8)		4705 (53.1)	810 (48.1)		15,248 (52.1)	13,717 (52.8)	1531 (45.2)	
Race/ethnicity, N (%)				< 0.001			< 0.001				< 0.001
Non-Hispanic White	5686 (75.8)	3834 (74.2)	1852 (79.4)		4680 (74.8)	1006 (80.9)		14,002 (72.8)	12,249 (72.3)	1753 (77.0)	
Non-Hispanic Black	1728 (8.8)	1268 (9.5)	460 (7.4)		1508 (9.3)	220 (6.4)		5538 (9.5)	4972 (9.6)	566 (8.5)	
Mexican American	1906 (6.2)	1390 (6.7)	516 (5.1)		1586 (6.4)	320 (5.0)		4401 (6.9)	4024 (7.2)	377 (4.0)	
Other	966 (9.2)	737 (9.6)	229 (8.1)		836 (9.5)	130 (7.6)		4572 (10.8)	4120 (10.8)	452 (10.5)	
Education, N (%)				< 0.001			< 0.001				< 0.001
Less than high school	2397 (14.3)	1632 (13.0)	765 (17.0)		1935 (13.7)	462 (16.7)		5877 (12.9)	5018 (12.3)	859 (18.2)	
High school or equivalent	2460 (24.7)	1628 (22.6)	832 (29.4)		2031 (24.1)	429 (27.4)		6600 (23.4)	5776 (22.8)	824 (28.6)	
College or above	5429 (61.0)	3969 (64.5)	1460 (53.6)		4644 (62.1)	785 (55.9)		16,036 (63.8)	14,571 (64.9)	1465 (53.2)	
Income-to-poverty ratio	3.4 (1.7, 5.0)	3.5 (1.8, 5.0)	3.0 (1.5, 4.8)	< 0.001	3.4 (1.8, 5.0)	3.2 (1.7, 4.9)	0.03	3.3 (1.7, 5.0)	3.4 (1.8, 5.0)	2.5 (1.2, 4.4)	< 0.001
Marital status, N (%)				< 0.001			< 0.001				< 0.001
Married or living with partner	6505 (66.3)	4542 (65.9)	1963 (67.2)		5410 (65.8)	1095 (68.3)		17,539 (65.1)	15,724 (65.5)	1815 (61.1)	
Never married	1829 (17.8)	1395 (19.3)	434 (14.5)		1610 (18.8)	219 (12.9)		5399 (18.5)	5052 (19.4)	347 (10.4)	
Widowed, divorced, or separated	1952 (15.9)	1292 (14.9)	660 (18.3)		1590 (15.3)	362 (18.8)		5575 (16.4)	4589 (15.1)	986 (28.5)	
History of cancer, N (%)				0.054			0.027				< 0.001
No	9454 (92.3)	6691 (92.7)	2763 (91.4)		7936 (92.6)	1518 (90.8)		26,030 (91.1)	23,387 (92.0)	2643 (82.9)	
Yes	832 (7.7)	538 (7.3)	294 (8.6)		674 (7.4)	158 (9.2)		2483 (8.9)	1978 (8.0)	505 (17.1)	
Body mass index, kg/m^2^	27.0 (23.7, 31.2)	26.8 (23.5, 30.9)	27.5 (24.0, 31.9)	< 0.001	27.0 (23.7, 31.1)	27.1 (23.5, 31.3)	0.785	27.4 (23.9, 31.7)	27.2 (23.8, 31.5)	28.9 (25.0, 33.9)	< 0.001
Waist circumference, cm	95.2 (85.1, 106.0)	94.3 (84.3, 105.2)	97.0 (86.5, 107.4)	< 0.001	95.2 (85.1, 106.1)	95.3 (84.9, 105.5)	0.668	96.5 (86.0, 107.4)	95.9 (85.6, 106.7)	102.0 (91.4, 113.4)	< 0.001
Abdominal obesity, N (%)				< 0.001			0.324				< 0.001
No	5081 (51.8)	3724 (53.8)	1357 (47.4)		4298 (52.1)	783 (50.3)		13,509 (48.2)	12,490 (49.9)	1019 (32.6)	
Yes	5205 (48.2)	3505 (46.2)	1700 (52.6)		4312 (47.9)	893 (49.7)		15,004 (51.8)	12,875 (50.1)	2129 (67.4)	
Hyperglycemia, N (%)				0.022			0.395				< 0.001
No	7927 (81.2)	5631 (82.0)	2296 (79.3)		6666 (81.3)	1261 (80.3)		20,833 (76.3)	18,890 (77.6)	1943 (64.2)	
Yes	2359 (18.8)	1598 (18.0)	761 (20.7)		1944 (18.7)	415 (19.7)		7680 (23.7)	6475 (22.4)	1205 (35.8)	
Hypertension, N (%)				< 0.001			0.352				< 0.001
No	6461 (67.9)	4685 (69.6)	1776 (64.4)		5451 (68.2)	1010 (66.8)		17,471 (66.1)	16,235 (68.5)	1236 (43.8)	
Yes	3825 (32.1)	2544 (30.4)	1281 (35.6)		3159 (31.8)	666 (33.2)		11,042 (33.9)	9130 (31.5)	1912 (56.2)	
Hyperlipidemia, N (%)				0.002			0.014				< 0.001
No	3023 (30.4)	2207 (31.7)	816 (27.7)		2576 (31.0)	447 (27.8)		9008 (32.2)	8334 (33.5)	674 (20.3)	
Yes	7263 (69.6)	5022 (68.3)	2241 (72.3)		6034 (69.0)	1229 (72.2)		19,505 (67.8)	17,031 (66.5)	2474 (79.7)	
Smoke, N (%)				< 0.001			< 0.001				< 0.001
No	7916 (76.5)	5714 (79.0)	2202 (71.2)		6713 (77.7)	1203 (70.8)		22,388 (78.8)	20,132 (79.6)	2256 (71.6)	
Yes	2370 (23.5)	1515 (21.0)	855 (28.8)		1897 (22.3)	473 (29.2)		6125 (21.2)	5233 (20.4)	892 (28.4)	
Excess alcohol intake, N (%)				0.122			0.422				< 0.001
No	6389 (60.1)	4498 (60.8)	1891 (58.5)		5346 (60.3)	1043 (58.9)		17,778 (59.4)	15,568 (58.5)	2210 (67.3)	
Yes	3897 (39.9)	2731 (39.2)	1166 (41.5)		3264 (39.7)	633 (41.1)		10,735 (40.6)	9797 (41.5)	938 (32.7)	
Unhealthy diet, N (%)				0.004			< 0.001				0.995
No	6137 (58.3)	4386 (59.6)	1751 (55.5)		5178 (59.2)	959 (54.3)		17,214 (59.4)	15,311 (59.4)	1903 (59.4)	
Yes	4149 (41.7)	2843 (40.4)	1306 (44.5)		3432 (40.8)	717 (45.7)		11,299 (40.6)	10,054 (40.6)	1245 (40.6)	
Physical inactivity, N (%)				< 0.001			< 0.001				0.001
No	3394 (30.7)	2584 (33.6)	810 (24.5)		2955 (32.1)	439 (24.3)		9568 (32.9)	8637 (33.3)	931 (29.3)	
Yes	6892 (69.3)	4645 (66.4)	2247 (75.5)		5655 (67.9)	1237 (75.7)		18,945 (67.1)	16,728 (66.7)	2217 (70.7)	
Metabolic status, N (%)				< 0.001			0.082				< 0.001
Good	1555 (17.3)	1176 (18.6)	379 (14.5)		1338 (17.8)	217 (15.1)		4270 (16.7)	4089 (17.8)	181 (6.6)	
Moderate	5739 (57.5)	4089 (57.9)	1650 (56.8)		4816 (57.4)	923 (58.4)		15,272 (55.0)	13,927 (56.2)	1345 (44.1)	
Poor	2992 (25.1)	1964 (23.5)	1028 (28.6)		2456 (24.8)	536 (26.5)		8971 (28.3)	7349 (26.0)	1622 (49.3)	
Lifestyle status, N (%)				< 0.001			< 0.001				0.165
Good	1099 (10.0)	853 (11.5)	246 (6.6)		972 (10.8)	127 (6.1)		2900 (9.8)	2608 (9.9)	292 (8.9)	
Moderate	7019 (67.2)	4984 (67.9)	2035 (65.7)		5889 (67.4)	1130 (66.5)		20,023 (69.5)	17,832 (69.6)	2191 (68.9)	
Poor	2168 (22.8)	1392 (20.5)	776 (27.7)		1749 (21.8)	419 (27.4)		5590 (20.7)	4925 (20.6)	665 (22.2)	
Metabolic-Lifestyle status, N (%)				< 0.001			< 0.001				< 0.001
Good-Good	144 (1.4)	123 (1.8)	21 (0.7)		126 (1.5)	18 (1.1)		442 (1.7)	425 (1.8)	17 (0.5)	
Good-Moderate	1017 (11.4)	778 (12.2)	239 (9.4)		887 (11.8)	130 (9.3)		2832 (11.2)	2732 (11.9)	100 (4.1)	
Good-Poor	394 (4.6)	275 (4.6)	119 (4.4)		325 (4.5)	69 (4.7)		996 (3.9)	932 (4.1)	64 (2.0)	
Moderate-Good	602 (5.8)	486 (7.0)	116 (3.4)		543 (6.5)	59 (2.8)		1556 (5.4)	1446 (5.6)	110 (3.7)	
Moderate-Moderate	3823 (37.6)	2763 (38.4)	1060 (35.7)		3216 (37.5)	607 (37.9)		10,539 (37.5)	9625 (38.4)	914 (29.0)	
Moderate-Poor	1314 (14.1)	840 (12.5)	474 (17.7)		1057 (13.4)	257 (17.7)		3177 (12.1)	2856 (12.2)	321 (11.4)	
Poor-Good	353 (2.7)	244 (2.8)	109 (2.6)		303 (2.8)	50 (2.2)		902 (2.7)	737 (2.4)	165 (4.8)	
Poor-Moderate	2179 (18.3)	1443 (17.3)	736 (20.5)		1786 (18.1)	393 (19.3)		6652 (20.8)	5475 (19.2)	1177 (35.8)	
Poor-Poor	460 (4.1)	277 (3.4)	183 (5.5)		367 (3.9)	93 (5.0)		1417 (4.8)	1137 (4.3)	280 (8.7)	

Data are presented as median (IQR) or N (%); absolute numbers were unweighted; medians, percentages, and IQR were estimated after weighted; P-values were obtained from nonparametric Wilcoxon rank-sum tests for continuous variables and from Rao-Scott adjusted chi-square tests for categorical variables

*Abbreviations*: *LBP* Low back pain, *NP* Neck pain, *BN*-*FL* Back- or neck-related functional limitation

In the functional limitation cohort, 28,513 adults were included, with a median age of 44 years. Among these, 3148 reported BN-FL. Compared to participants without BN-FL, those with BN-FL were older and had a higher proportion of abdominal obesity, hyperglycemia, hypertension, dyslipidemia, smoking, and physical inactivity, but a lower proportion of excess alcohol intake (Table 1). Characteristics of participants with sedentary time are presented in Supplementary Table S2.

### Prevalence of LBP, NP, and BN-FL across individual metabolic and lifestyle status

**Fig. 2:**
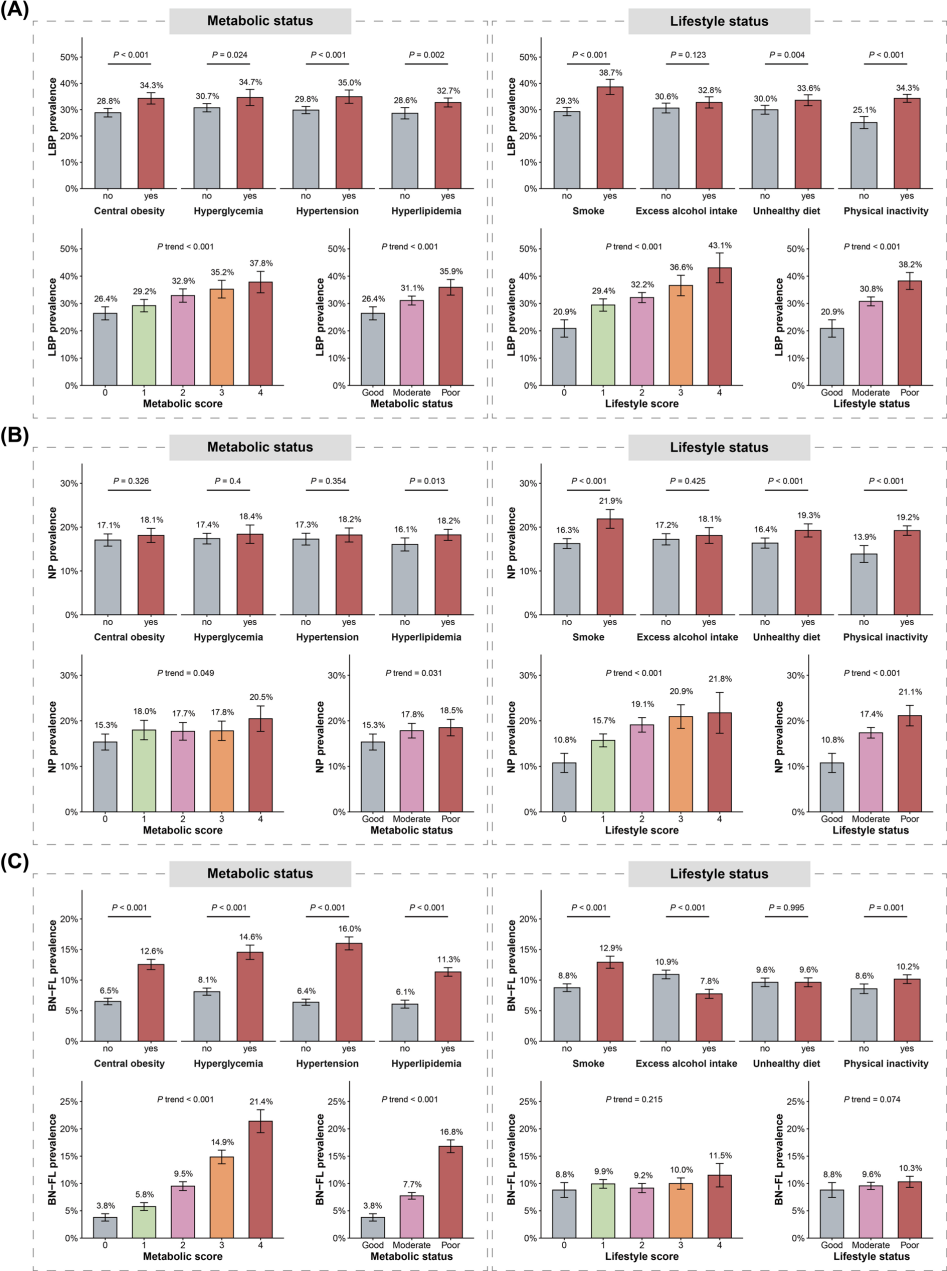
Weighted prevalence of outcomes grouped by individual metabolic health and lifestyle. **A** LBP prevalence; **B **NP prevalence; **C** BN-FL prevalence. Metabolic and lifestyle scores were calculated as the count of risk factors present. For each score, participants were classified into three categories: good (0), moderate (1–2), and poor (3–4). Abbreviations: LBP, low back pain; NP, neck pain; BN-FL, back- or neck-related functional limitation

The prevalence of LBP was higher in all metabolic and lifestyle exposome groups (all *p* < 0.05), except for those with excess alcohol intake, compared to the non-exposome group (Fig. 2A). As metabolic and lifestyle scores increased, prevalence showed a clear linear trend (both *p* trend < 0.001). It ranged from 26.4% in individuals with good metabolic status to 35.9% in those with poor metabolic status (*p* trend < 0.001), and from 20.9% in individuals with good lifestyle status to 38.2% in those with poor lifestyle status (*p* trend < 0.001).

The prevalence of NP was higher in the hyperlipidemia (*p* = 0.013), smoking (*p* < 0.001), unhealthy diet (*p* < 0.001), and physical inactivity (*p* < 0.001) groups compared to the non-exposome group (Fig. 2B). As metabolic and lifestyle scores increased, prevalence also showed a clear linear increase (both *p* trend < 0.05), ranging from 15.3% in individuals with good metabolic status to 18.5% in those with poor metabolic status (*p* trend = 0.031), and from 10.8% in individuals with good lifestyle status to 21.1% in those with poor lifestyle status (*p* trend < 0.001).

The prevalence of BN-FL was higher in each metabolic factor group (all *p* < 0.001), as well as the smoking (*p* < 0.001) and physical inactivity (*p* = 0.001) groups, compared to the non-exposome group (Fig. 2C). However, it was lower in individuals with excess alcohol intake (*p* < 0.001). As the metabolic score increased, a clear linear increase in prevalence was observed, ranging from 3.8% in individuals with good metabolic status to 16.8% in those with poor metabolic status (*p* trend < 0.001).

### Prevalence of LBP, NP, and BN-FL across combined metabolic and lifestyle status

**Fig. 3:**
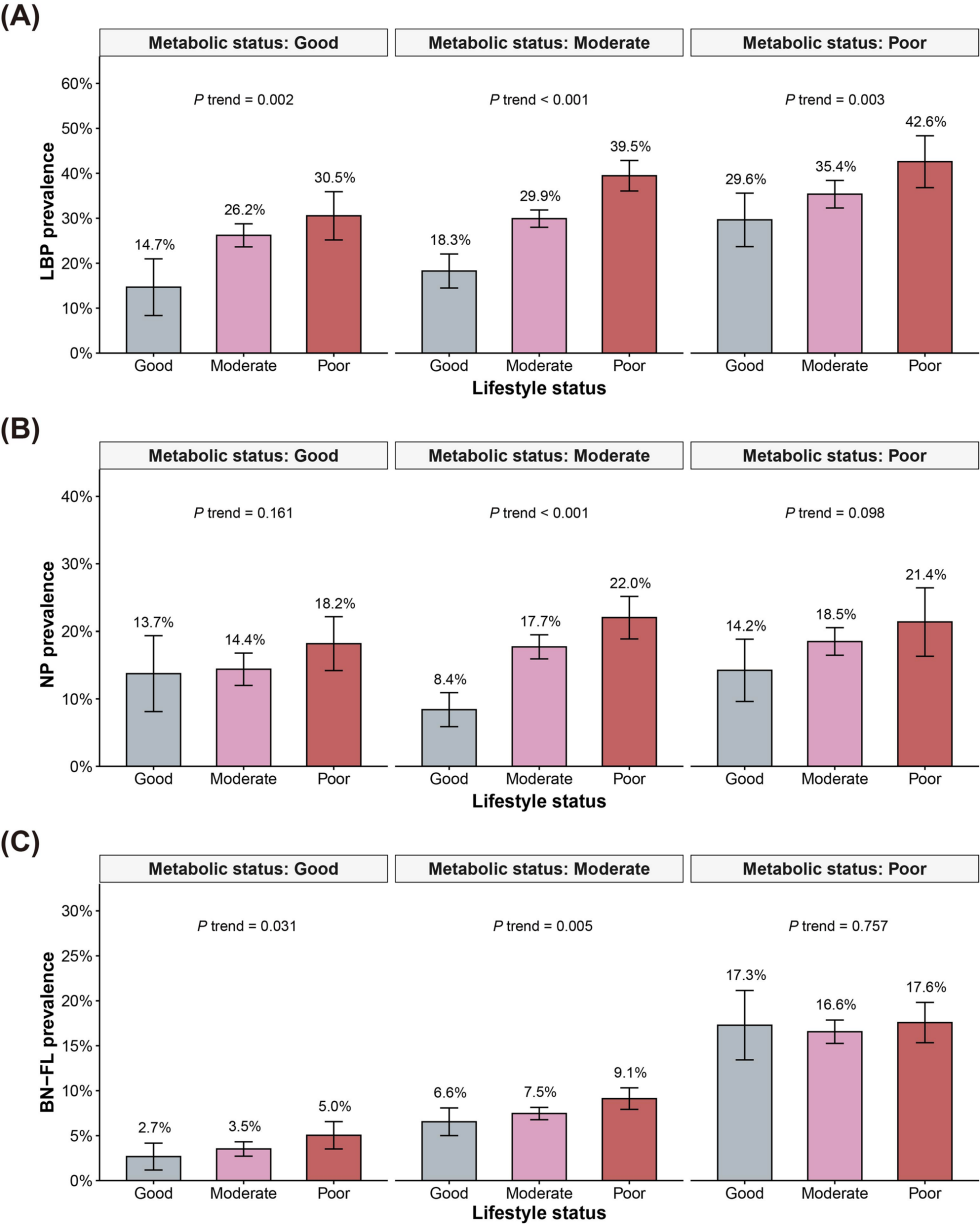
Weighted prevalence of outcomes grouped by combined metabolic health and lifestyle. **A** LBP prevalence; **B** NP prevalence; **C** BN-FL prevalence. Metabolic and lifestyle scores were calculated as the count of risk factors present. For each score, participants were classified into three categories: good (0), moderate (1–2), and poor (3–4). Abbreviations: LBP, low back pain; NP, neck pain; BN-FL, back- or neck-related functional limitation

Regardless of metabolic status, the prevalence of LBP showed a clear increasing trend with the deterioration of lifestyle status (all *p* trend < 0.05), reaching 42.6% in individuals with poor metabolic-lifestyle status (Fig. 3A). For NP, the prevalence increased with worsening lifestyle status only in individuals with moderate metabolic status (*p* trend < 0.001), peaking at 22.0% in those with moderate metabolic health and poor lifestyle (Fig. 3B). For BN-FL, the prevalence increased with worsening lifestyle status in individuals with good and moderate metabolic status (both *p* trend < 0.05) (Fig. 3C).

### Individual associations of metabolic and lifestyle status with LBP, NP, and BN-FL

**Fig. 4:**
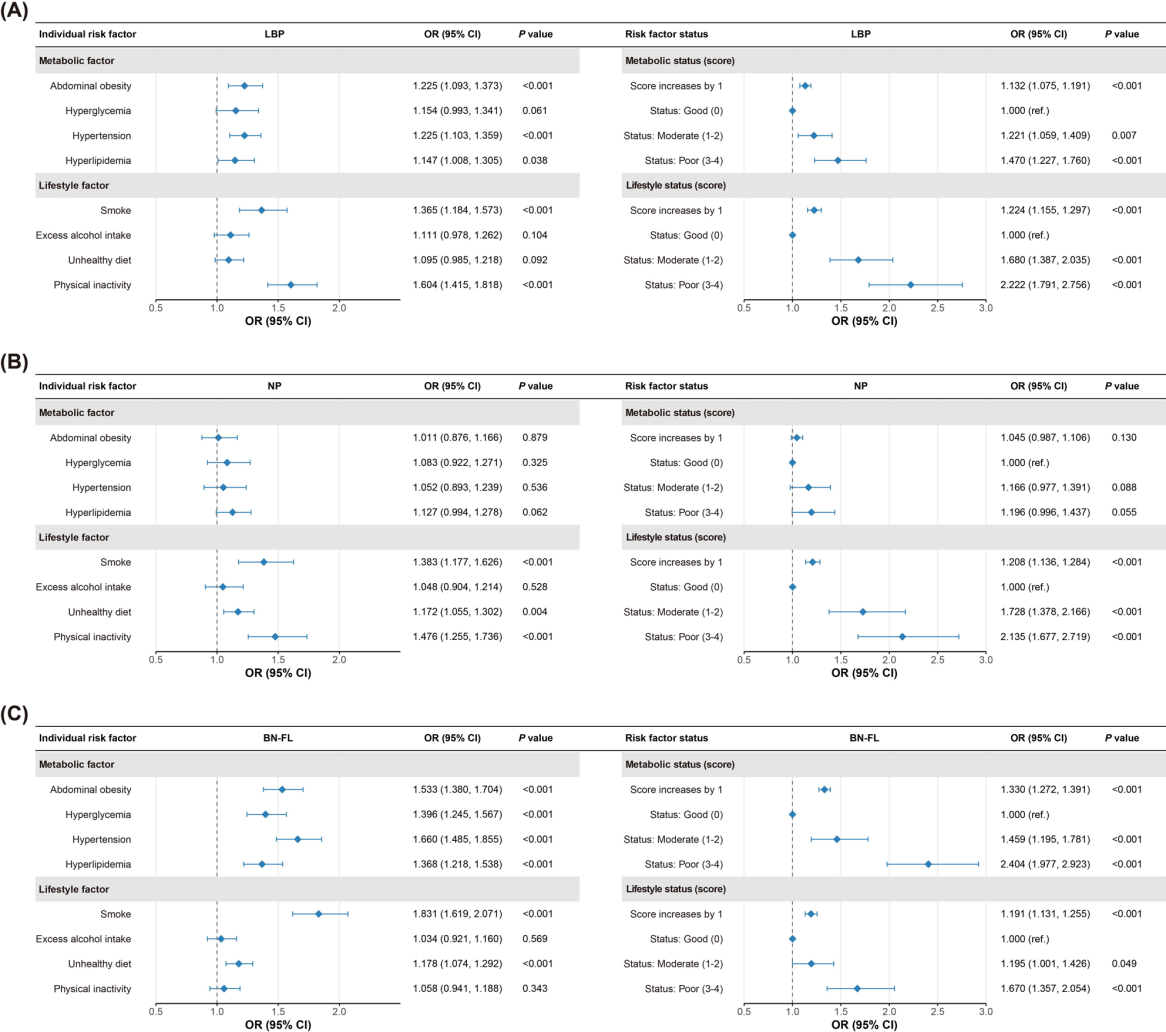
Individual associations of metabolic health and lifestyle with outcomes. **A** LBP; **B** NP; **C** BN-FL. Metabolic and lifestyle scores were calculated as the count of risk factors present. For each score, participants were classified into three categories: good (0), moderate (1–2), and poor (3–4). Models were adjusted for age, sex, race/ethnicity, income, education, marital status, and history of cancer/malignancy. Abbreviations: LBP, low back pain; NP, neck pain; BN-FL, back- or neck-related functional limitation

Multivariable logistic regression revealed that abdominal obesity, hypertension, dyslipidemia, smoking, and physical inactivity were associated with higher odds of LBP. Individuals with poor metabolic status had higher odds of LBP (OR = 1.470, 95% CI: 1.227–1.760) compared to those with good metabolic status. Similarly, poor lifestyle status was associated with higher odds of LBP (OR = 2.222, 95% CI: 1.791–2.756) (Fig. 4A). In sensitivity analyses, additional adjustment for metabolic and lifestyle factors yielded similar results, although dyslipidemia was no longer associated (Supplementary Figure S2A). PAF analysis showed that lifestyle factors were associated with higher estimated contributions to LBP, with physical inactivity showing the largest estimated PAF ( 20.8%) (Supplementary Figure S3A).

Metabolic status was not associated with NP (Fig. 4B). Lifestyle factors such as smoking, unhealthy diet, and physical inactivity were associated with NP, with poor lifestyle status showing higher odds (OR = 2.135, 95% CI: 1.677–2.719) compared to good lifestyle status. In sensitivity analyses, the results remained consistent (Supplementary Figure S2B). Physical inactivity had the highest PAF of 20.5% (Supplementary Figure S3B).

All metabolic factors, as well as lifestyle factors such as smoking and an unhealthy diet, were associated with BN-FL. Individuals with poor metabolic status had higher odds of BN-FL (OR = 2.404, 95% CI: 1.977–2.923) compared to those with good metabolic status. Similarly, poor lifestyle status was associated with higher odds of BN-FL (OR = 1.670, 95% CI: 1.357–2.054) (Fig. 4C). In sensitivity analyses, unhealthy diet was no longer associated with BN-FL (Supplementary Figure S2C). Abdominal obesity had the highest PAF of 15.1%, followed by smoking (14.0%) and hypertension (12.6%) (Supplementary Figure S3C).

### Combined associations of metabolic and lifestyle status with LBP, NP, and BN-FL

**Fig. 5:**
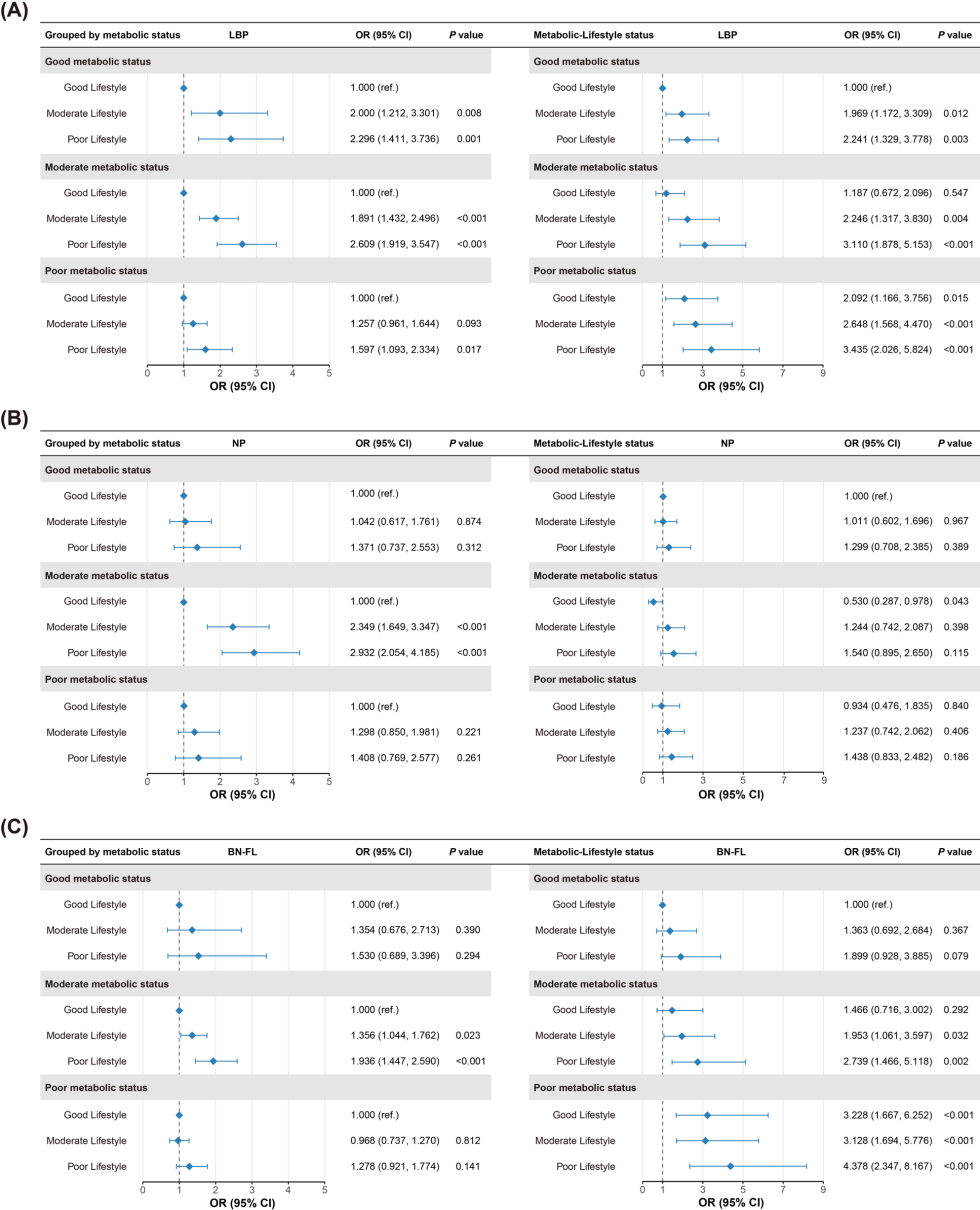
Combined associations of metabolic health and lifestyle with outcomes. **A** LBP; **B** NP; **C** BN-FL. Left panels (Grouped by metabolic status) present associations between lifestyle status and outcomes within each metabolic status category, using participants with good lifestyle as the reference in each stratum. Right panels (Metabolic-lifestyle status) present joint analyses across nine combined categories, using participants with both good metabolic and lifestyle status as the reference. Models were adjusted for age, sex, race/ethnicity, income, education, marital status, and history of cancer/malignancy. Abbreviations: LBP, low back pain; NP, neck pain; BN-FL, back- or neck-related functional limitation

For LBP, worsening lifestyle status was consistently associated with higher odds across all levels of metabolic status (Fig. 5A). Compared with individuals with both good metabolic and good lifestyle status, those with both poor metabolic and poor lifestyle status had substantially higher odds of LBP (OR = 3.435, 95% CI: 2.026–5.824). Subgroup analyses revealed stronger associations among younger adults (OR = 6.008, 95% CI: 2.935–12.300) and males (OR = 4.135, 95% CI: 1.802–9.488) (Supplementary Figure S4 and S5).

Worsening lifestyle status was positively associated with NP in individuals with moderate metabolic status (Fig. 5B). Subgroup analyses revealed that individuals with both poor metabolic and poor lifestyle status had higher odds of NP among younger adults (OR = 2.321, 95% CI: 1.242–4.340) compared with those with good metabolic and lifestyle status (Supplementary Figure S6 and S7).

For BN-FL, worsening lifestyle status was significantly associated with higher odds among individuals with moderate metabolic status (Fig. 5C). Participants with both poor metabolic and poor lifestyle status exhibited the highest odds of BN-FL (OR = 4.378, 95% CI: 2.347–8.167). These associations persisted across young and middle-aged individuals, both sexes, and both sedentary and non-sedentary populations (Supplementary Figure S8-S10), with a stronger association observed among non-sedentary participants (OR = 6.401, 95% CI: 2.479–16.531).

In addition, similar patterns were observed when weighted metabolic and lifestyle scores were applied (Supplementary Figure S11), with generally consistent associations across outcomes. These findings indicate robustness to alternative scoring approaches and support the use of the simpler unweighted scoring method in practice.

## Discussion

### Main findings

This nationally representative study demonstrated significant associations of metabolic and lifestyle profiles with LBP and BN-FL, whereas only lifestyle profiles were associated with NP. Individuals with both poor metabolic and poor lifestyle status had higher odds of LBP compared with those with good status, suggesting a potential combined association across these domains. These associations appeared more evident among younger adults and men. Lifestyle factors, especially physical inactivity, showed the largest estimated PAFs for LBP and NP, while metabolic factors, notably abdominal obesity, showed the largest estimated PAFs for BN-FL. However, given the cross-sectional design, these estimates should be interpreted cautiously, as the observed associations do not establish temporality and reverse causality cannot be excluded. Overall, these findings underscore the potential value of considering both metabolic and lifestyle profiles in risk stratification of low back or neck pain and related disability.

### Practical implications

The combined associations of lifestyle behaviors observed in this study align with previous findings [21]. This study showed that physical inactivity and smoking were the most prominent correlates. Physical inactivity may be linked to deconditioning of trunk and neck musculature, impaired postural control, and systemic inflammation, all of which are associated with persistent pain [10, 22]. Previous studies have shown that individuals with greater smoking exposure experience higher pain severity and disability, likely due to smoking’s reduction of spinal blood flow and its acceleration of intervertebral disc degeneration [23, 24]. In contrast, unhealthy diet showed an association only with NP, and alcohol consumption was not associated with any outcome. Notably, excess alcohol consumption was associated with a lower prevalence of BN-FL and a negative PAF estimate, which should be interpreted with caution. A likely explanation is reverse causality, as individuals with functional limitations or pain may reduce or avoid alcohol consumption, either due to health concerns or reduced social engagement, given that alcohol use is often a social behavior [25]. Residual confounding may also contribute, as overall health status or other unmeasured behaviors could influence both alcohol use and outcomes. Although some prior studies have reported a potential inverse relationship between moderate alcohol intake and pain symptoms, findings remain inconsistent [26, 27]. Importantly, these observations should not be interpreted as evidence of a protective effect of alcohol consumption.

Among metabolic factors, abdominal obesity showed the strongest associations with LBP and BN-FL. Visceral adiposity has been linked to chronic low-grade inflammation through the release of adipokines and cytokines, which may sensitize nociceptive pathways and relate to tissue degeneration [28]. Additionally, mechanical overload from central adiposity increases spinal stress, further exacerbating pain symptoms. These mechanisms partly explain our observation that poor metabolic status was associated with LBP and BN-FL, but not with NP. A prospective longitudinal cohort study found that individuals who adopt optimal lifestyle behaviors, such as adequate physical activity, maintaining an ideal BMI, and not smoking, experience less activity limitation from LBP [29], which further supports causality and strengthens our findings.

Understanding individual characteristics can help tailor interventions for musculoskeletal pain [30]. While unhealthy lifestyle behaviors and metabolic abnormalities are recognized risk factors for musculoskeletal pain [9, 31–33], there is limited evidence on how to identify individuals who would benefit most from lifestyle interventions based on their metabolic health. In this study, individuals with unhealthy lifestyles exhibited significantly higher odds of LBP across all metabolic status, and exhibited higher odds of NP and BN-FL among those with mildly compromised metabolic health. These findings suggest that considering metabolic status alongside lifestyle behaviors may aid in risk stratification of musculoskeletal pain and related disability.

Prolonged sedentary behavior, increasingly common in modern societies, is linked to higher risks of musculoskeletal pain, including LBP and NP [32–35]. Due to the limited modifiability of sedentary behavior in occupational and transport settings, identifying other modifiable risk factors is crucial. In this study, poor metabolic-lifestyle status was associated with BN-FL in both sedentary and non-sedentary groups, highlighting the potential value of targeting metabolic and lifestyle factors in managing back and neck problems.

The Lancet Series has emphasized the need to prioritize LBP, alongside other musculoskeletal conditions, as a major public health issue [4]. They advocate for a “living well” approach to LBP, promoting person-centered care that emphasizes self-management and healthy lifestyles to restore function and optimize participation [4]. Lifestyle modifications are also prioritized in managing metabolic syndrome [15]. The limited research on self-management for LBP and the ineffectiveness of current trials may stem from insufficient consideration of individual characteristics and inadequate targeting of risk factors [36, 37]. Moreover, clinical care standards currently lack specific recommendations for individuals with LBP [38]. Therefore, we call for a stronger focus on integrating lifestyle modifications with metabolic disease management to alleviate pain, improve function, and more effectively address individual needs.

### Strengths and limitations

This study has several strengths. First, it used a large, nationally representative dataset spanning 20 years. Second, it assessed LBP, NP, and BN-FL, covering outcomes from pain to functional impact. Functional limitation represents a broader dimension of disease burden beyond pain symptoms alone, reflecting the long-term consequences of chronic LBP or NP on daily functioning and quality of life. Third, it was the first study to examine the associations of lifestyle factors with LBP, NP, and BN-FL across different metabolic statuses, as well as the combined associations of metabolic health and lifestyle on outcomes. Finally, the analysis included populations of varying ages, genders, and sedentary behaviors, confirming the generalizability of the results.

However, several limitations should be considered. First, the cross-sectional design limited the ability to establish causal relationships, and reverse causality may have occurred as pain and functional limitations could have affected lifestyle behaviors. Second, reliance on self-reported data introduced potential recall bias. Third, dietary intake was assessed using a single 24-hour recall, which may not fully represent participants’ usual dietary patterns, likely biasing the observed associations toward the null. In addition, the use of distribution-based thresholds to define unhealthy diet and physical inactivity may limit comparability across populations and may not fully align with clinically established cutoffs. Fourth, the wide confidence intervals for the risk estimates related to poor combined metabolic-lifestyle status indicated some uncertainty. Fifth, due to sample size limitations and lack of data on sedentary time, sensitivity analyses were not performed in the pain cohort. Sixth, potential confounders such as mental health conditions, occupational physical workload, sleep quality, comorbidities, and medication use were not accounted for. Mental health can influence physical activity and pain perception, occupational workload may contribute to musculoskeletal strain, poor sleep can increase pain sensitivity [39, 40], and comorbidities and medication use may affect both metabolic status and pain outcomes, potentially biasing the observed lifestyle-pain associations.

### Future research

Future research should focus on longitudinal studies to establish causal relationships, refine interventions targeting lifestyle and metabolic health, and explore personalized strategies for preventing and managing low back and neck problems.

## Conclusion

Our study identified consistent associations between lifestyle factors and LBP across metabolic statuses, with a graded increase in risk across combined metabolic-lifestyle categories. Both metabolic and lifestyle profiles were associated with LBP and BN-FL, while only lifestyle profiles were associated with NP. These findings emphasize the importance of considering both metabolic and lifestyle status in the risk stratification of LBP and related disabilities and point to the potential value of targeting modifiable factors. Future longitudinal studies are needed to establish temporality and explore strategies targeting lifestyle and metabolic health in the context of musculoskeletal pain.

## Supplementary information

Supplementary Material 1.

## Data Availability

The datasets generated and/or analysed during the current study are available in the NHANES repository, https://wwwn.cdc.gov/nchs/nhanes/.

## References

[1] Collaborators GLBP. Global, regional, and national burden of low back pain, 1990–2020, its attributable risk factors, and projections to 2050: a systematic analysis of the Global Burden of Disease Study 2021. Lancet Rheumatol. 2023;5(6):e316–29. 37273833 10.1016/S2665-9913(23)00098-X PMC10234592

[2] Safiri S, Kolahi AA, Hoy D, Buchbinder R, Mansournia MA, Bettampadi D et al. Global, regional, and national burden of neck pain in the general population, 1990–2017: systematic analysis of the Global Burden of Disease Study 2017. BMJ (Clinical research ed). 2020;368:m791. 10.1136/bmj.m791 PMC7249252 32217608

[3] Pocovi NC, Elkins MR. Low back pain management. J Physiotherapy. 2025;71(2):78–80. 10.1016/j.jphys.2025.03.008 40175232

[4] Buchbinder R, van Tulder M, Öberg B, Costa LM, Woolf A, Schoene M, et al. Low back pain: a call for action. Lancet. 2018;391(10137):2384–8. 29573871 10.1016/S0140-6736(18)30488-4

[5] The Lancet R. The global epidemic of low back pain. Lancet Rheumatol. 2023;5(6):e305. 10.1016/S2665-9913(23)00133-9 38251593

[6] Wong CK, Mak RY, Kwok TS, Tsang JS, Leung MY, Funabashi M, et al. Prevalence, incidence, and factors associated with non-specific chronic low back pain in community-dwelling older adults aged 60 years and older: a systematic review and meta-analysis. J pain. 2022;23(4):509–34. 34450274 10.1016/j.jpain.2021.07.012

[7] Luc A, Antoine F, Bekkering G, Detrembleur C, Pitance L. Relationship between leisure time physical activity, weight, and the onset and persistence of nonspecific neck pain: a systematic review. J Orthop Sports Phys Ther. 2022;52(12):777–91. 35960506 10.2519/jospt.2022.11137

[8] Mäntyselkä P, Kautiainen H, Vanhala M. Prevalence of neck pain in subjects with metabolic syndrome–a cross-sectional population-based study. BMC Musculoskelet Disord. 2010;11:171. 20670458 10.1186/1471-2474-11-171 PMC2918543

[9] Yan W, Kong L, He T, Guo G, Zhu Q, Xi X, Fang M. Association between metabolic syndrome and low back pain: a two-sample Mendelian randomization study. Sci Rep. 2025;15(1):17686. 40399540 10.1038/s41598-025-02630-7 PMC12095482

[10] Damato TM, Christofaro DGD, Pinheiro MB, Morelhao PK, Pinto RZ, De Oliveira Silva D, et al. Does sedentary behaviour contribute to the development of a new episode of low back pain? A systematic review of prospective cohort studies. Eur J Pain. 2022;26(7):1412–23. 35598285 10.1002/ejp.1977

[11] Li M, Xu Y, Wan Q, Shen F, Xu M, Zhao Z, et al. Individual and combined associations of modifiable lifestyle and metabolic health status with new-onset diabetes and major cardiovascular events: The China Cardiometabolic Disease and Cancer Cohort (4 C) Study. Diabetes Care. 2020;43(8):1929–36. 32540923 10.2337/dc20-0256

[12] Wertli MM, Held U, Campello M, Schecter Weiner S. Obesity is associated with more disability at presentation and after treatment in low back pain but not in neck pain: findings from the OIOC registry. BMC Musculoskelet Disord. 2016;17:140. 10.1186/s12891-016-0992-0 PMC4815184 27036857

[13] Coppock JA, Danyluk ST, Englander ZA, Spritzer CE, Goode AP, DeFrate LE. Increasing BMI increases lumbar intervertebral disc deformation following a treadmill walking stress test. J Biomech. 2021;121:110392. 33819699 10.1016/j.jbiomech.2021.110392 PMC8128153

[14] Popescu A, Lee H. Neck pain and lower back pain. Med Clin North Am. 2020;104(2):279–92. 32035569 10.1016/j.mcna.2019.11.003

[15] Neeland IJ, Lim S, Tchernof A, Gastaldelli A, Rangaswami J, Ndumele CE, et al. Metabolic syndrome. Nat reviews Disease primers. 2024;10(1):77. 39420195 10.1038/s41572-024-00563-5

[16] Li Q, Huang X, Lu Y, Du X, Xuan Q. Individual and joint associations of modifiable metabolic status and lifestyle with kidney stones among sedentary population: insights from a nationally representative dataset. Int J Surg. 2026;112(1):178–89. 40961176 10.1097/JS9.0000000000003417 PMC12825901

[17] Zhang YB, Chen C, Pan XF, Guo J, Li Y, Franco OH, et al. Associations of healthy lifestyle and socioeconomic status with mortality and incident cardiovascular disease: two prospective cohort studies. BMJ. 2021;373:n604. 33853828 10.1136/bmj.n604 PMC8044922

[18] Chang Q, Zhu Y, Liu Z, Cheng J, Liang H, Lin F et al. Replacement of sedentary behavior with various physical activities and the risk of all-cause and cause-specific mortality. BMC Med. 2024;22(1):385. 10.1186/s12916-024-03599-2 PMC11395964 39267013

[19] Wang X, Tian L, Xu Q, Pan X, Kong L, Zhao H. Association between metabolism and low back pain: a cross-sectional study. J Orthop Surg Res. 2025;20(1):784. 40841650 10.1186/s13018-025-06218-9 PMC12369122

[20] Xie S, Xiao H, Li G, Zheng J, Zhang F, Lan Y, Luo M. Association between a body shape index and low back pain: a cross-sectional study highlighting gender-specific differences in NHANES data. BMC Public Health. 2025;25(1):753. 39994591 10.1186/s12889-025-21904-3 PMC11852558

[21] Skillgate E, Pico-Espinosa OJ, Hallqvist J, Bohman T, Holm L. Healthy lifestyle behavior and risk of long duration troublesome neck pain or low back pain among men and women: results from the Stockholm Public Health Cohort. Clin Epidemiol. 2017;9:491–500. 29066933 10.2147/CLEP.S145264 PMC5644563

[22] Bergens O, Nilsson A, Papaioannou KG, Kadi F. Sedentary patterns and systemic inflammation: sex-specific links in older adults. Front Physiol. 2021;12:625950. 33613317 10.3389/fphys.2021.625950 PMC7892961

[23] Schönnagel L, Hoehl BU, Taheri N, Becker L, Suwalski P, Schömig F, et al. The impact of smoking on low back pain and disability. Eur J Pain. 2025;29(6):e70041. 40392623 10.1002/ejp.70041

[24] Hendrix J, Nijs J, Ickmans K, Godderis L, Ghosh M, Polli A. The interplay between oxidative stress, exercise, and pain in health and disease: potential role of autonomic regulation and epigenetic mechanisms. Antioxid (Basel Switzerland). 2020;9(11):1166. 10.3390/antiox9111166 PMC7700330 33238564

[25] Cunha Ferro R, de Oliveira PSG, Teixeira RP. Wine consumption: an exploratory study on hospitality and commensality. Int J Gastronomy Food Sci. 2025;41:101263.

[26] Skillgate E, Vingård E, Josephson M, Holm LW, Alfredsson L. Is smoking and alcohol consumption associated with long-term sick leave due to unspecific back or neck pain among employees in the public sector? Results of a three-year follow-up cohort study. J Rehabil Med. 2009;41(7):550–6. 19543666 10.2340/16501977-0370

[27] Lv Z, Cui J, Zhang J. Smoking, alcohol and coffee consumption and risk of low back pain: a Mendelian randomization study. Eur Spine J. 2022;31(11):2913–9. 36114324 10.1007/s00586-022-07389-3

[28] Binvignat M, Sellam J, Berenbaum F, Felson DT. The role of obesity and adipose tissue dysfunction in osteoarthritis pain. Nat Rev Rheumatol. 2024;20(9):565–84. 39112603 10.1038/s41584-024-01143-3

[29] Roberts KE, Beckenkamp PR, Ferreira ML, Ho EK, Carvalho ESAP, Calais-Ferreira L, Ferreira PH. The impact of aggregate positive lifestyle behaviors on low back pain resilience and care seeking. Spine J. 2023;23(10):1405–13. 37393016 10.1016/j.spinee.2023.06.388

[30] Cashin AG, Sterling M. Optimising physical rehabilitation for people with musculoskeletal pain. Pain. 2025;166(11S):S131–5. 41086344 10.1097/j.pain.0000000000003719

[31] Meert L, Picavet HSJ, Vervullens S, Meeus M, Van Kuijk SMJ, Verschuren WMM, Smeets R. Exploring the association of metabolic factors and chronic musculoskeletal pain over a period of 10 years - the Doetinchem Cohort Study. Clin Rheumatol. 2025;44(2):839–53. 39694973 10.1007/s10067-024-07251-5

[32] Mazaheri-Tehrani S, Arefian M, Abhari AP, Riahi R, Vahdatpour B, Baradaran Mahdavi S, Kelishadi R. Sedentary behavior and neck pain in adults: a systematic review and meta-analysis. Prev Med. 2023;175:107711. 10.1016/j.ypmed.2023.107711 37775083

[33] Dzakpasu FQS, Carver A, Brakenridge CJ, Cicuttini F, Urquhart DM, Owen N, Dunstan DW. Musculoskeletal pain and sedentary behaviour in occupational and non-occupational settings: a systematic review with meta-analysis. Int J Behav Nutr Phys Activity. 2021;18(1):159. 10.1186/s12966-021-01191-y PMC8666269 34895248

[34] Matthews CE, Carlson SA, Saint-Maurice PF, Patel S, Salerno EA, Loftfield E, et al. Sedentary behavior in U.S. adults: fall 2019. Med Sci Sports Exerc. 2021;53(12):2512–9. 34310489 10.1249/MSS.0000000000002751 PMC8595506

[35] Kallings LV, Blom V, Ekblom B, Holmlund T, Eriksson JS, Andersson G et al. Workplace sitting is associated with self-reported general health and back/neck pain: a cross-sectional analysis in 44,978 employees. BMC Public Health. 2021;21(1):875. 10.1186/s12889-021-10893-8 PMC8101162 33957889

[36] Maher C, Lin C-WC. Welcome new evidence on self-management of back pain. Lancet Rheumatol. 2024;6(7):e412–3. 38824936 10.1016/S2665-9913(24)00116-4

[37] Denneny D, Walumbe J. Physical activity to prevent recurrences of low back pain. Lancet. 2024;404(10448):98–100. 38908391 10.1016/S0140-6736(24)01247-9

[38] Oliveira CB, Machado GC, Underwood M, Maher CG. NICE Standard for low back pain and sciatica needs urgent revision. Br J Sports Med. 2025;59(13):884–7. 40280731 10.1136/bjsports-2025-109817

[39] Joensen EDR, Frederiksen L, Frederiksen SV, Valeur ES, Giordano R, Hertel E, Petersen KK. Sex and sleep quality effects on the relationship between sleep disruption and pain sensitivity. Eur J Pain. 2025;29(5):e70023. 40197999 10.1002/ejp.70023 PMC11977682

[40] Scaini S, Davies S, De Francesco S, Pelucchi A, Rubino S, Battaglia M. Altered pain perception and nociceptive thresholds in major depression and anxiety disorders: a meta-analysis. Neurosci Biobehav Rev. 2025;169:106014. 39828235 10.1016/j.neubiorev.2025.106014

