# Supplementary material for: Individual and combined associations of modifiable metabolic health and lifestyle with low back pain, neck pain, and functional limitation: evidence from a nationally cross-sectional study
Source: BMC Public Health. 2026 May 1;26:1902. doi: 10.1186/s12889-026-27651-3 (PMC13277017; doi:10.1186/s12889-026-27651-3)
Supplement: Supplementary file 1 — Supplementary Material 1. [file 12889_2026_27651_MOESM1_ESM.docx]

**Supplementary Material**

**Individual and Combined Associations of Modifiable Metabolic Health and Lifestyle with Low Back Pain, Neck Pain, and Functional Limitation: Evidence from a Nationally Cross-sectional Study**

Chengxin Xie, Qihang Li, Jingxin Xin, Xiao Jiang, Qiang He, Wenbo Bian, Dongting Yu, Yanru Lin, Zhenyu Yao

**Supplementary Table S1** Physical function tasks assessing functional limitations in the NHANES structured questionnaires

**Supplementary Table S2** Characteristics of the study population with sedentary time from functional limitation cohort

**Supplementary Figure S1** Frequency distribution of participants by metabolic and lifestyle score categories

**Supplementary Figure S2** Individual associations of metabolic health and lifestyle with outcomes: sensitivity analyses

**Supplementary Figure S3** Population attributable fractions for outcomes associated with individual metabolic and lifestyle risk components

**Supplementary Figure S4** Combined associations of metabolic health and lifestyle with low back pain: subgroup analyses by age

**Supplementary Figure S5** Combined associations of metabolic health and lifestyle with low back pain: subgroup analyses by sex

**Supplementary Figure S6** Combined associations of metabolic health and lifestyle with neck pain: subgroup analyses by age

**Supplementary Figure S7** Combined associations of metabolic health and lifestyle with neck pain: subgroup analyses by sex

**Supplementary Figure S8** Combined associations of metabolic health and lifestyle with back- or neck-related functional limitation: subgroup analyses by age

**Supplementary Figure S9** Combined associations of metabolic health and lifestyle with back- or neck-related functional limitation: subgroup analyses by sex

**Supplementary Figure S10** Combined associations of metabolic health and lifestyle with back- or neck-related functional limitation: sensitivity analyses by sedentary behavior

**Supplementary Figure S11** Combined associations of metabolic health and lifestyle with outcomes: sensitivity analyses by weighted scoring

**Supplementary Table S1** Physical function tasks assessing functional limitations in the NHANES structured questionnaires

| Codes | Items | Questions |
| --- | --- | --- |
| *The next questions ask about difficulties you may have doing certain activities because of a health problem. By "health problem" we mean any long-term physical, mental or emotional problem or illness {not including pregnancy}. By yourself and without using any special equipment, how much difficulty do you have . . .* | | |
| PFQ061A | Managing money difficulty | . . .managing your money (such as keeping track of your expenses or paying bills)? |
| PFQ061B | Walking for a quarter mile difficulty | . . .walking for a quarter of a mile (that is about 2 or 3 blocks)? |
| PFQ061C | Walking up ten steps difficulty | . . .walking up 10 steps without resting? |
| PFQ061D | Stooping, crouching, kneeling difficulty | . . .stooping, crouching, or kneeling? |
| PFQ061E | Lifting or carrying difficulty | . . .lifting or carrying something as heavy as 10 pounds (like a sack of potatoes or rice)? |
| PFQ061F | House chore difficulty | . . .doing chores around the house (like vacuuming, sweeping, dusting, or straightening up)? |
| PFQ061G | Preparing meals difficulty | . . .preparing your own meals? |
| PFQ061H | Walking between rooms on same floor | . . .walking from one room to another on the same level? |
| PFQ061I | Standingup from armless chair difficulty | . . .standing up from an armless straight chair? |
| PFQ061J | Getting in and out of bed difficulty | . . .getting in or out of bed? |
| PFQ061K | Using fork, knife, drinking from cup | . . .eating, like holding a fork, cutting food or drinking from a glass? |
| PFQ061L | Dressing yourself difficulty | . . .dressing yourself, including tying shoes, working zippers, and doing buttons? |
| PFQ061M | Standing for long periods difficulty | . . .standing or being on your feet for about 2 hours? |
| PFQ061N | Sitting for long periods difficulty | . . .sitting for about 2 hours? |
| PFQ061O | Reaching up over head difficulty | . . .reaching up over your head? |
| PFQ061P | Grasp/holding small objects difficulty | . . .using your fingers to grasp or handle small objects? |
| PFQ061Q | Going out to movies, events difficulty | . . .going out to things like shopping, movies, or sporting events? |
| PFQ061R | Attending social event difficulty | . . .participating in social activities (visiting friends, attending clubs or meetings or going to parties)? |
| PFQ061S | Leisure activity at home difficulty | . . .doing things to relax at home or for leisure (reading, watching TV, sewing, listening to music)? |
| PFQ061T | Push or pull large objects difficulty | . . .pushing or pulling large objects like a living room chair? |
| *Participants who reported difficulty with any functional activities were then asked about the conditions or health problems that cause them to have difficulty or need help and were given the option to report up to 5 health problems:* | | |
| PFQ063A | Health problems causing difficulty | What condition or health problem causes you to have difficulty with or need help with (NAME OF UP TO 3 ACTIVITIES/these activities)? |
| PFQ063B |  |  |
| PFQ063C |  |  |
| PFQ063D |  |  |
| PFQ063E |  |  |

**Supplementary Table S2** Characteristics of the study population with sedentary time from functional limitation cohort

| Characteristics | Overall  (N = 18302) | BN-FL | | *P* value |
| --- | --- | --- | --- | --- |
|  |  | No  (N = 16249) | Yes  (N = 2053) |  |
| Age, year | 45 (31, 58) | 43 (31, 56) | 59 (46, 68) | < 0.001 |
| Age group, N (%) |  |  |  | < 0.001 |
| 20-44 years | 8612 (49.2) | 8183 (52.1) | 429 (22.7) |  |
| 45-64 years | 6287 (36.1) | 5414 (35.4) | 873 (42.8) |  |
| ≥ 65 years | 3403 (14.7) | 2652 (12.6) | 751 (34.5) |  |
| Sex, N (%) |  |  |  | < 0.001 |
| Female | 8549 (47.6) | 7479 (46.7) | 1070 (55.8) |  |
| Male | 9753 (52.4) | 8770 (53.3) | 983 (44.2) |  |
| Race/ethnicity, N (%) |  |  |  | < 0.001 |
| Non-Hispanic White | 8220 (70.3) | 7146 (69.8) | 1074 (74.6) |  |
| Non-Hispanic Black | 3698 (9.9) | 3307 (10.0) | 391 (9.3) |  |
| Mexican American | 2528 (7.6) | 2316 (7.9) | 212 (4.6) |  |
| Other | 3856 (12.2) | 3480 (12.3) | 376 (11.6) |  |
| Education, N (%) |  |  |  | < 0.001 |
| Less than high school | 3546 (12.2) | 2997 (11.6) | 549 (17.8) |  |
| High school or equivalent | 4096 (22.2) | 3560 (21.5) | 536 (28.6) |  |
| College or above | 10660 (65.7) | 9692 (67.0) | 968 (53.6) |  |
| Income-to-poverty ratio | 3.3 (1.6, 5.0) | 3.4 (1.7, 5.0) | 2.3 (1.1, 4.3) | < 0.001 |
| Marital status, N (%) |  |  |  | < 0.001 |
| Married or living with partner | 10986 (63.9) | 9839 (64.4) | 1147 (59.4) |  |
| Never married | 3738 (19.9) | 3479 (20.7) | 259 (11.9) |  |
| Widowed, divorced, or separated | 3578 (16.3) | 2931 (14.9) | 647 (28.7) |  |
| History of cancer, N (%) |  |  |  | < 0.001 |
| No | 16700 (90.5) | 14998 (91.5) | 1702 (81.0) |  |
| Yes | 1602 (9.5) | 1251 (8.5) | 351 (19.0) |  |
| Body mass index, kg/m^2^ | 27.6 (24.0, 32.0) | 27.5 (23.9, 31.8) | 29.3 (25.0, 34.2) | < 0.001 |
| Waist circumference, cm | 97.1 (86.5, 108.1) | 96.5 (86.1, 107.5) | 102.9 (91.9, 114.4) | < 0.001 |
| Abdominal obesity, N (%) |  |  |  | < 0.001 |
| No | 8474 (46.4) | 7828 (48.0) | 646 (31.0) |  |
| Yes | 9828 (53.6) | 8421 (52.0) | 1407 (69.0) |  |
| Hyperglycemia, N (%) |  |  |  | < 0.001 |
| No | 12972 (73.4) | 11764 (74.9) | 1208 (59.9) |  |
| Yes | 5330 (26.6) | 4485 (25.1) | 845 (40.1) |  |
| Hypertension, N (%) |  |  |  | < 0.001 |
| No | 11252 (66.0) | 10481 (68.6) | 771 (41.4) |  |
| Yes | 7050 (34.0) | 5768 (31.4) | 1282 (58.6) |  |
| Hyperlipidemia, N (%) |  |  |  | < 0.001 |
| No | 5970 (33.2) | 5523 (34.5) | 447 (20.7) |  |
| Yes | 12332 (66.8) | 10726 (65.5) | 1606 (79.3) |  |
| Smoke, N (%) |  |  |  | < 0.001 |
| No | 14488 (80.6) | 13029 (81.6) | 1459 (71.7) |  |
| Yes | 3814 (19.4) | 3220 (18.4) | 594 (28.3) |  |
| Excess alcohol intake, N (%) |  |  |  | < 0.001 |
| No | 11173 (58.2) | 9751 (57.3) | 1422 (66.5) |  |
| Yes | 7129 (41.8) | 6498 (42.7) | 631 (33.5) |  |
| Unhealthy diet, N (%) |  |  |  | 0.724 |
| No | 11114 (60.4) | 9884 (60.4) | 1230 (60.0) |  |
| Yes | 7188 (39.6) | 6365 (39.6) | 823 (40.0) |  |
| Physical inactivity, N (%) |  |  |  | < 0.001 |
| No | 8674 (48.1) | 7858 (48.9) | 816 (41.1) |  |
| Yes | 9628 (51.9) | 8391 (51.1) | 1237 (58.9) |  |
| Metabolic status, N (%) |  |  |  | < 0.001 |
| Good | 2752 (16.5) | 2635 (17.6) | 117 (6.4) |  |
| Moderate | 9608 (53.8) | 8766 (55.2) | 842 (41.6) |  |
| Poor | 5942 (29.7) | 4848 (27.3) | 1094 (51.9) |  |
| Lifestyle status, N (%) |  |  |  | 0.004 |
| Good | 2579 (14.2) | 2328 (14.4) | 251 (12.6) |  |
| Moderate | 12777 (69.6) | 11370 (69.8) | 1407 (68.0) |  |
| Poor | 2946 (16.1) | 2551 (15.8) | 395 (19.4) |  |
| Metabolic-Lifestyle status, N (%) |  |  |  | < 0.001 |
| Good-Good | 410 (2.5) | 395 (2.7) | 15 (0.7) |  |
| Good-Moderate | 1855 (11.2) | 1793 (12.0) | 62 (3.7) |  |
| Good-Poor | 487 (2.8) | 447 (2.8) | 40 (2.0) |  |
| Moderate-Good | 1396 (7.9) | 1298 (8.2) | 98 (5.3) |  |
| Moderate-Moderate | 6612 (37.0) | 6044 (38.0) | 568 (27.5) |  |
| Moderate-Poor | 1600 (9.0) | 1424 (9.0) | 176 (8.8) |  |
| Poor-Good | 773 (3.8) | 635 (3.5) | 138 (6.5) |  |
| Poor-Moderate | 4310 (21.5) | 3533 (19.9) | 777 (36.7) |  |
| Poor-Poor | 859 (4.4) | 680 (4.0) | 179 (8.7) |  |
| Sedentary time, h/day | 5.0 (4.0, 8.0) | 5.0 (3.0, 8.0) | 6.0 (4.0, 8.0) | 0.989 |
| Sedentary time, N (%) |  |  |  | 0.014 |
| < 5 h/day | 7938 (39.3) | 7093 (39.4) | 845 (38.1) |  |
| 5-8 h/day | 4914 (27.2) | 4295 (26.8) | 619 (31.0) |  |
| ≥ 8 h/day | 5450 (33.5) | 4861 (33.8) | 589 (30.9) |  |
| Abbreviations: BN-FL, Back- or Neck-related Functional Limitation.  Data are presented as median (IQR) or N (%); absolute numbers were unweighted; medians, percentages, and IQR were estimated after weighted; *P*-values were obtained from nonparametric Wilcoxon rank-sum tests for continuous variables and from Rao-Scott adjusted chi-square tests for categorical variables. | | | | |


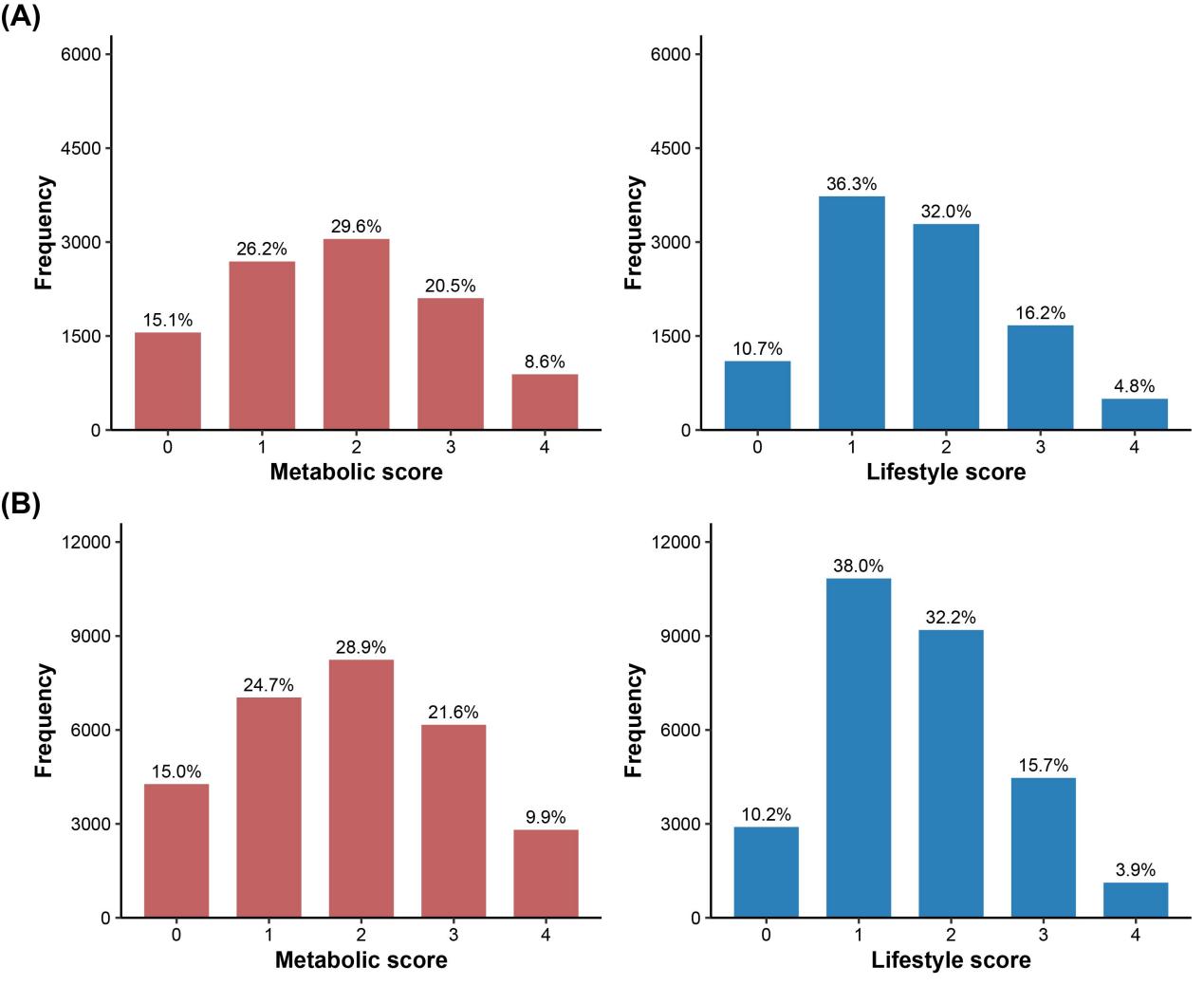


**Supplementary Figure S1** Frequency distribution of participants by metabolic and lifestyle score categories. (A ) pain cohort; (B) functional limitation cohort.

Percentages were weighted estimates.

The score of 0 was defined as the reference group (Good), representing participants with no metabolic or lifestyle risk factors. Due to the relatively low frequency of participants with a score of 4, scores of 3 and 4 were combined into a high-risk group (Poor). Scores of 1 and 2 were classified as the moderate-risk group (Moderate).


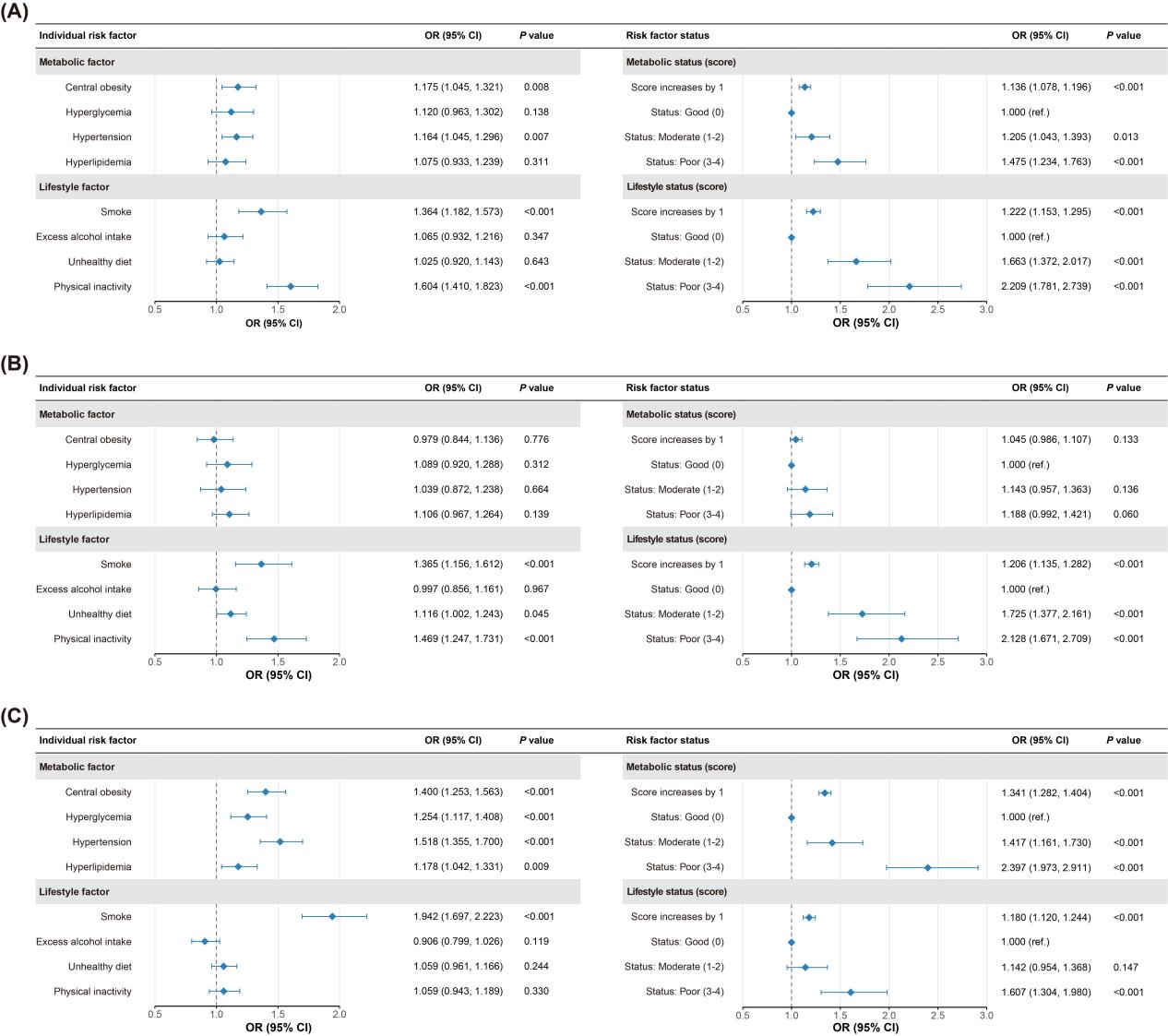


**Supplementary Figure S2** Individual associations of metabolic health and lifestyle with outcomes: sensitivity analyses. (A) low back pain; (B) neck pain; (C) back- or neck-related functional limitation.

Metabolic and lifestyle scores were calculated as the count of risk factors present. For each score, participants were classified into three categories: good (0), moderate (1-2), and poor (3-4).

Models were adjusted for age, sex, race/ethnicity, income, education, marital status, history of cancer/malignancy, and other metabolic and lifestyle risk components.


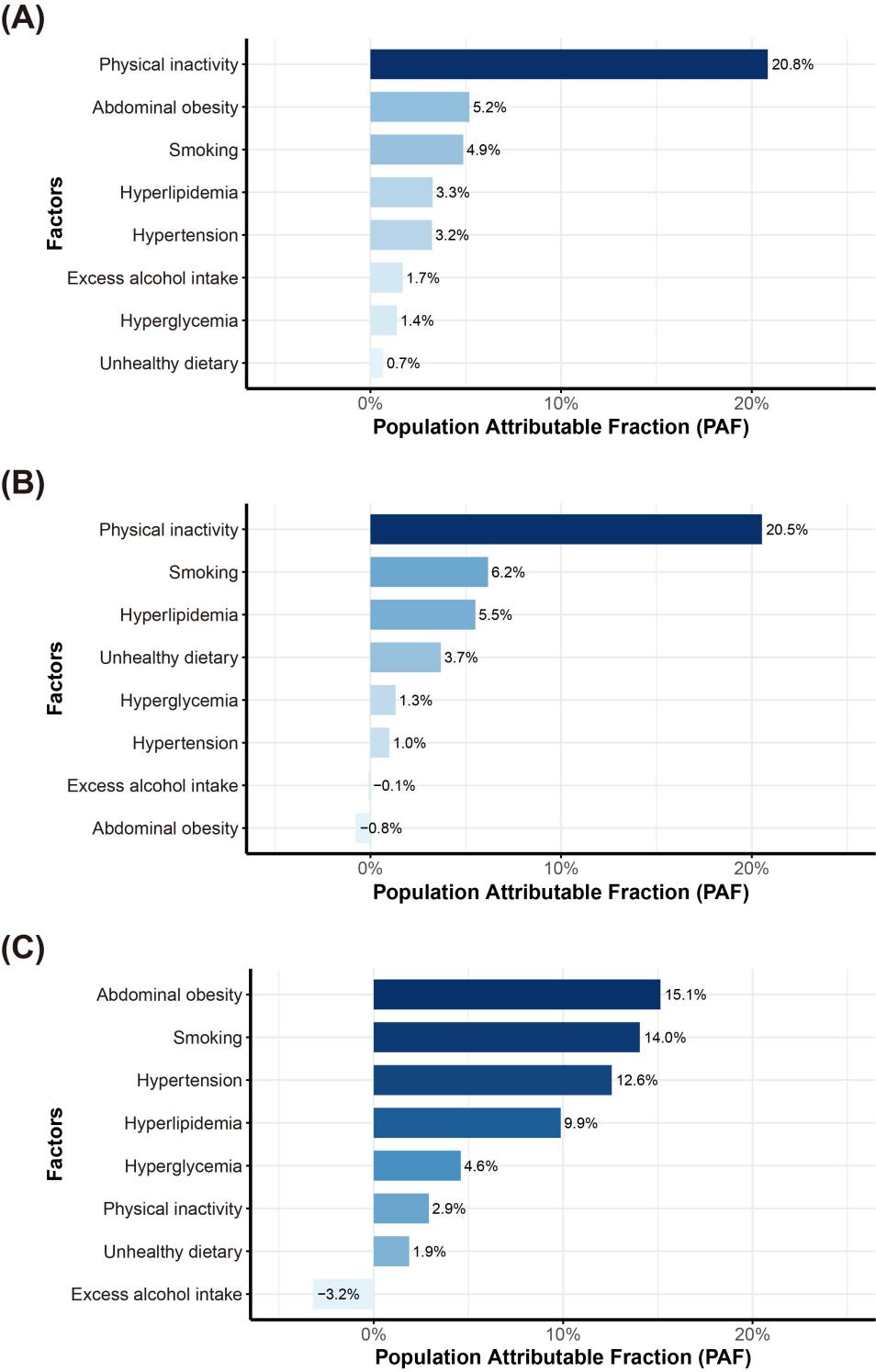


**Supplementary Figure S3** Population attributable fractions for outcomes associated with individual metabolic and lifestyle risk components. (A) low back pain; (B) neck pain; (C) back- or neck-related functional limitation.

Models were adjusted for age, sex, race/ethnicity, income, education, marital status, history of cancer/malignancy, and other metabolic and lifestyle risk components.

**
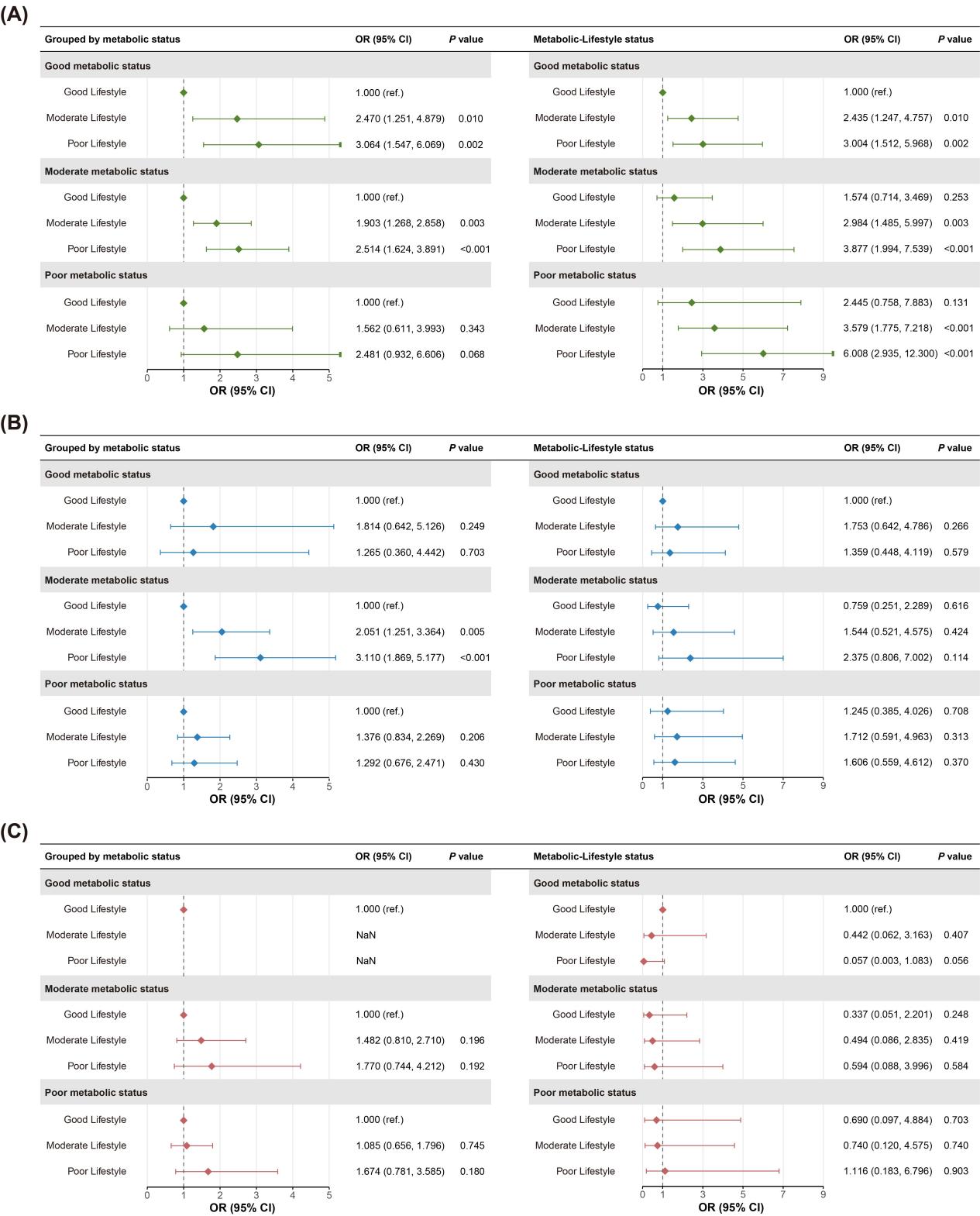
**

**Supplementary Figure S4** Combined associations of metabolic health and lifestyle with low back pain: subgroup analyses by age. (A) 20-44 years old; (B) 45-64 years old; (C) ≥ 65 years old.

Left panels present associations between lifestyle status and outcomes within each metabolic status category. Right panels present joint analyses across nine combined metabolic-lifestyle categories, using participants with both good metabolic and lifestyle status as the reference group.

Models were adjusted for age, sex, race/ethnicity, income, education, marital status, and history of cancer/malignancy.


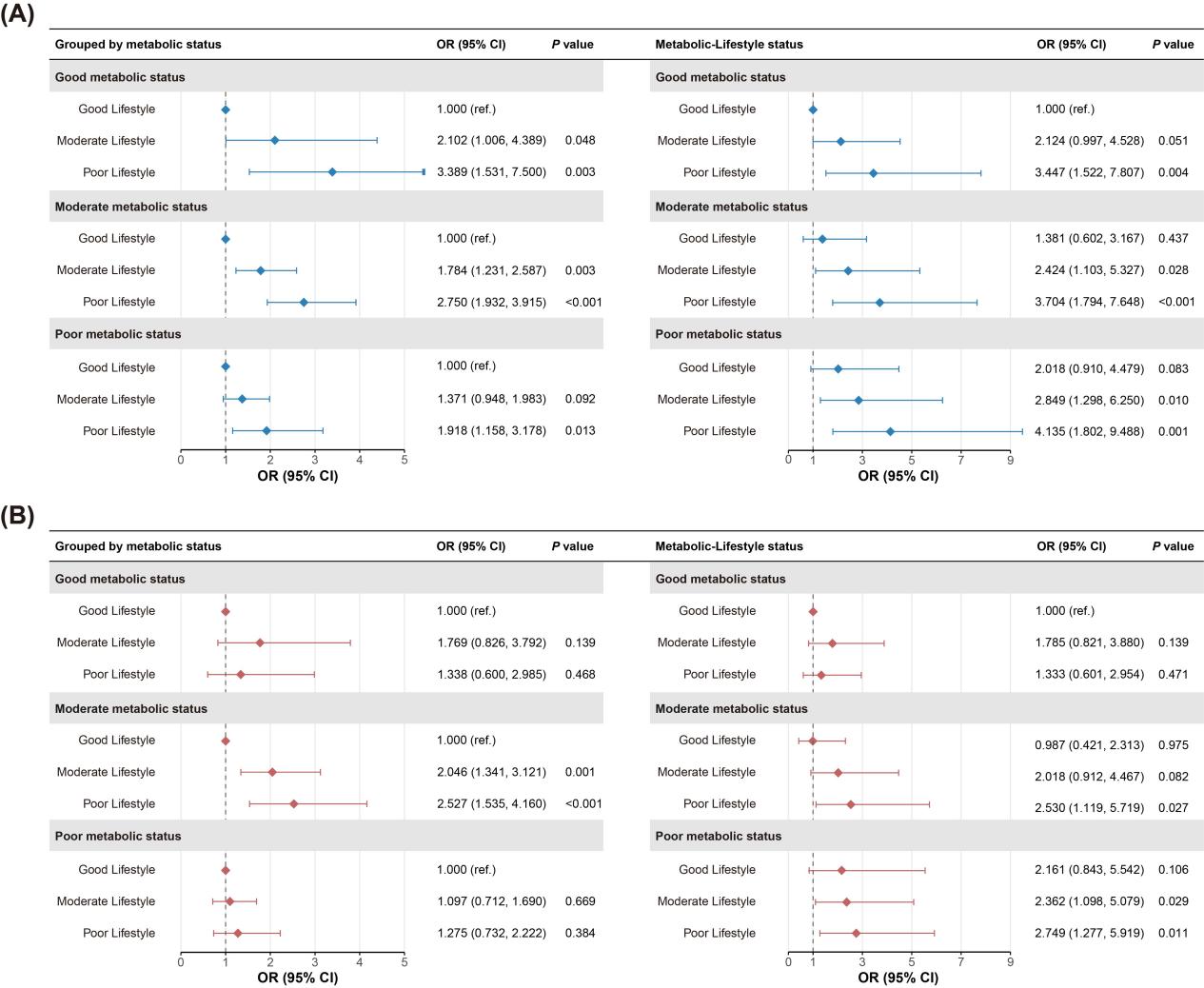


**Supplementary Figure S5** Combined associations of metabolic health and lifestyle with low back pain: subgroup analyses by sex. (A) male; (B) female.

Left panels present associations between lifestyle status and outcomes within each metabolic status category. Right panels present joint analyses across nine combined metabolic-lifestyle categories, using participants with both good metabolic and lifestyle status as the reference group.

Models were adjusted for age, race/ethnicity, income, education, marital status, and history of cancer/malignancy.


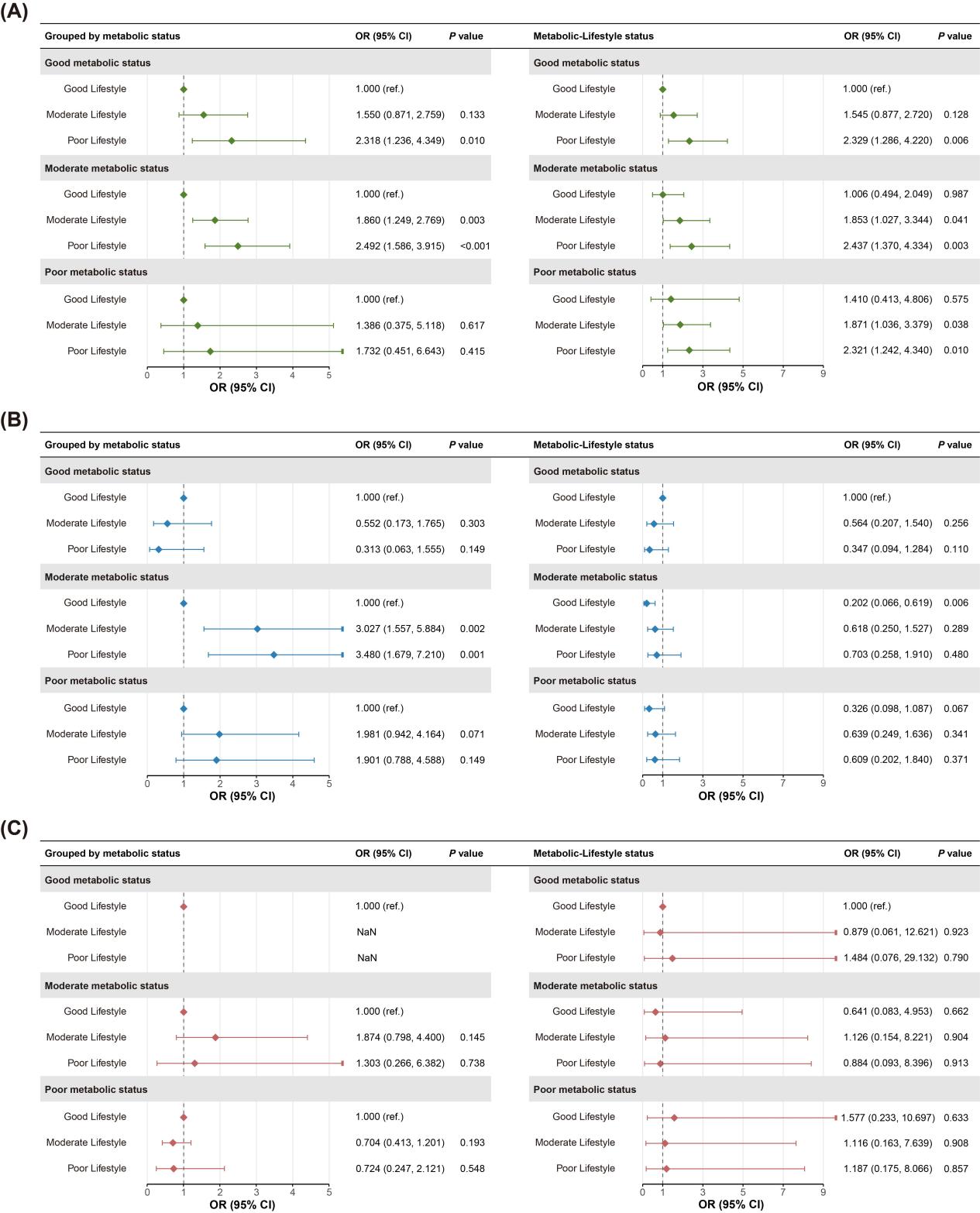


**Supplementary Figure S6** Combined associations of metabolic health and lifestyle with neck pain: subgroup analyses by age. (A) 20-44 years old; (B) 45-64 years old; (C) ≥ 65 years old.

Left panels present associations between lifestyle status and outcomes within each metabolic status category. Right panels present joint analyses across nine combined metabolic-lifestyle categories, using participants with both good metabolic and lifestyle status as the reference group.

Models were adjusted for age, sex, race/ethnicity, income, education, marital status, and history of cancer/malignancy.


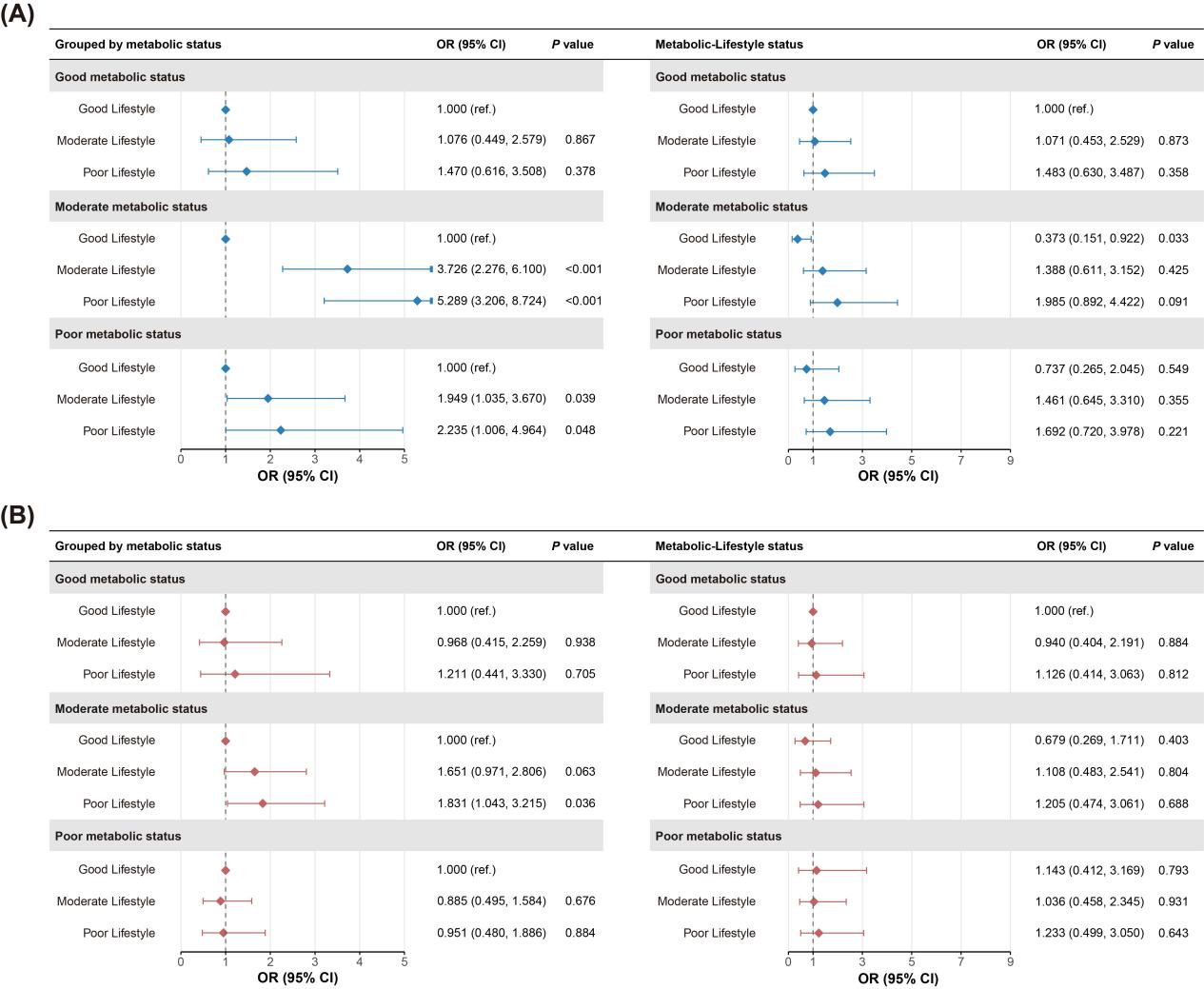


**Supplementary Figure S7** Combined associations of metabolic health and lifestyle with neck pain: subgroup analyses by sex. (A) male; (B) female.

Left panels present associations between lifestyle status and outcomes within each metabolic status category. Right panels present joint analyses across nine combined metabolic-lifestyle categories, using participants with both good metabolic and lifestyle status as the reference group.

Models were adjusted for age, race/ethnicity, income, education, marital status, and history of cancer/malignancy.


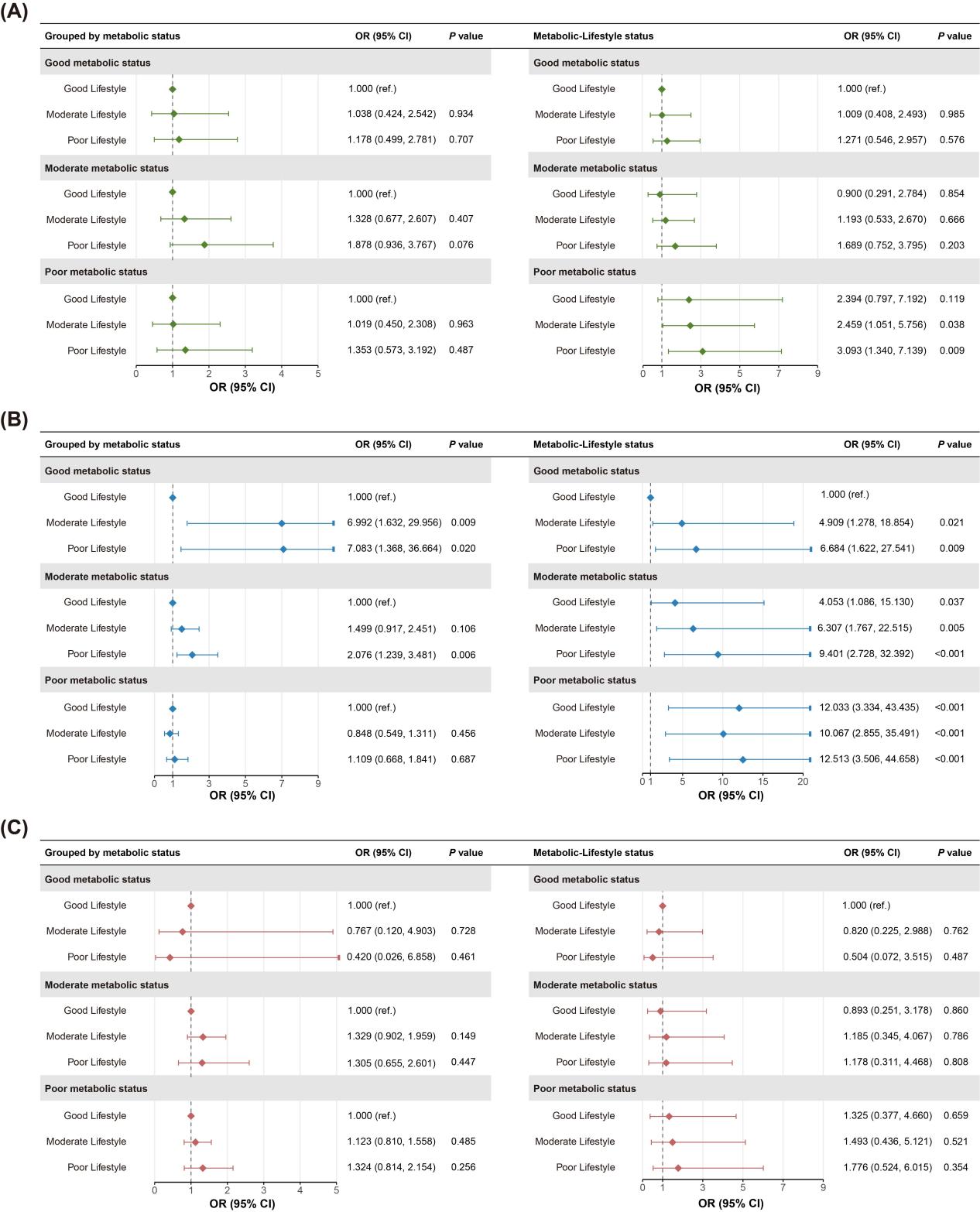


**Supplementary Figure S8** Combined associations of metabolic health and lifestyle with back- or neck-related functional limitation: subgroup analyses by age. (A) 20-44 years old; (B) 45-64 years old; (C) ≥ 65 years old.

Left panels present associations between lifestyle status and outcomes within each metabolic status category. Right panels present joint analyses across nine combined metabolic-lifestyle categories, using participants with both good metabolic and lifestyle status as the reference group.

Models were adjusted for age, sex, race/ethnicity, income, education, marital status, and history of cancer/malignancy.


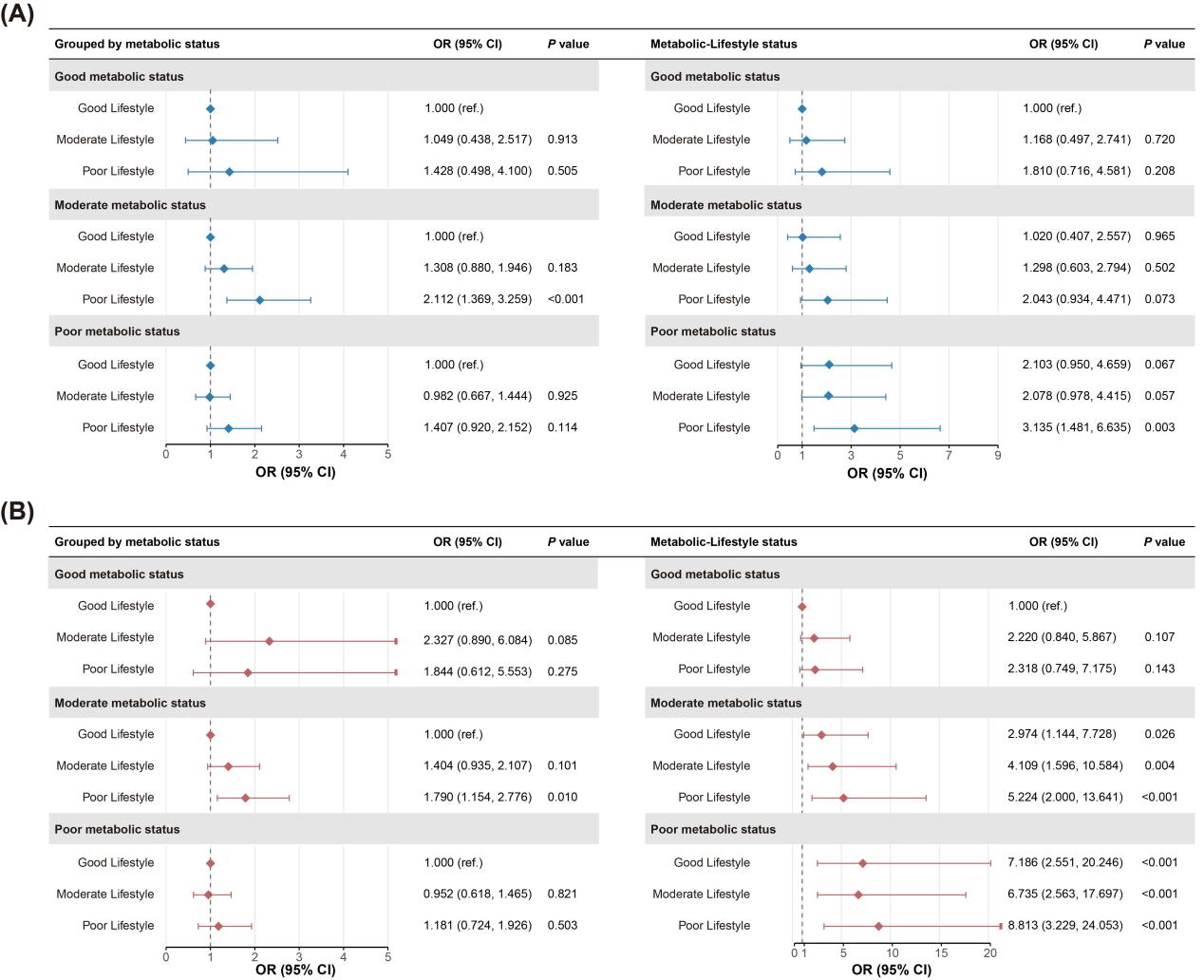


**Supplementary Figure S9** Combined associations of metabolic health and lifestyle with back- or neck-related functional limitation: subgroup analyses by sex. (A) male; (B) female.

Left panels present associations between lifestyle status and outcomes within each metabolic status category. Right panels present joint analyses across nine combined metabolic-lifestyle categories, using participants with both good metabolic and lifestyle status as the reference group.

Models were adjusted for age, race/ethnicity, income, education, marital status, and history of cancer/malignancy.


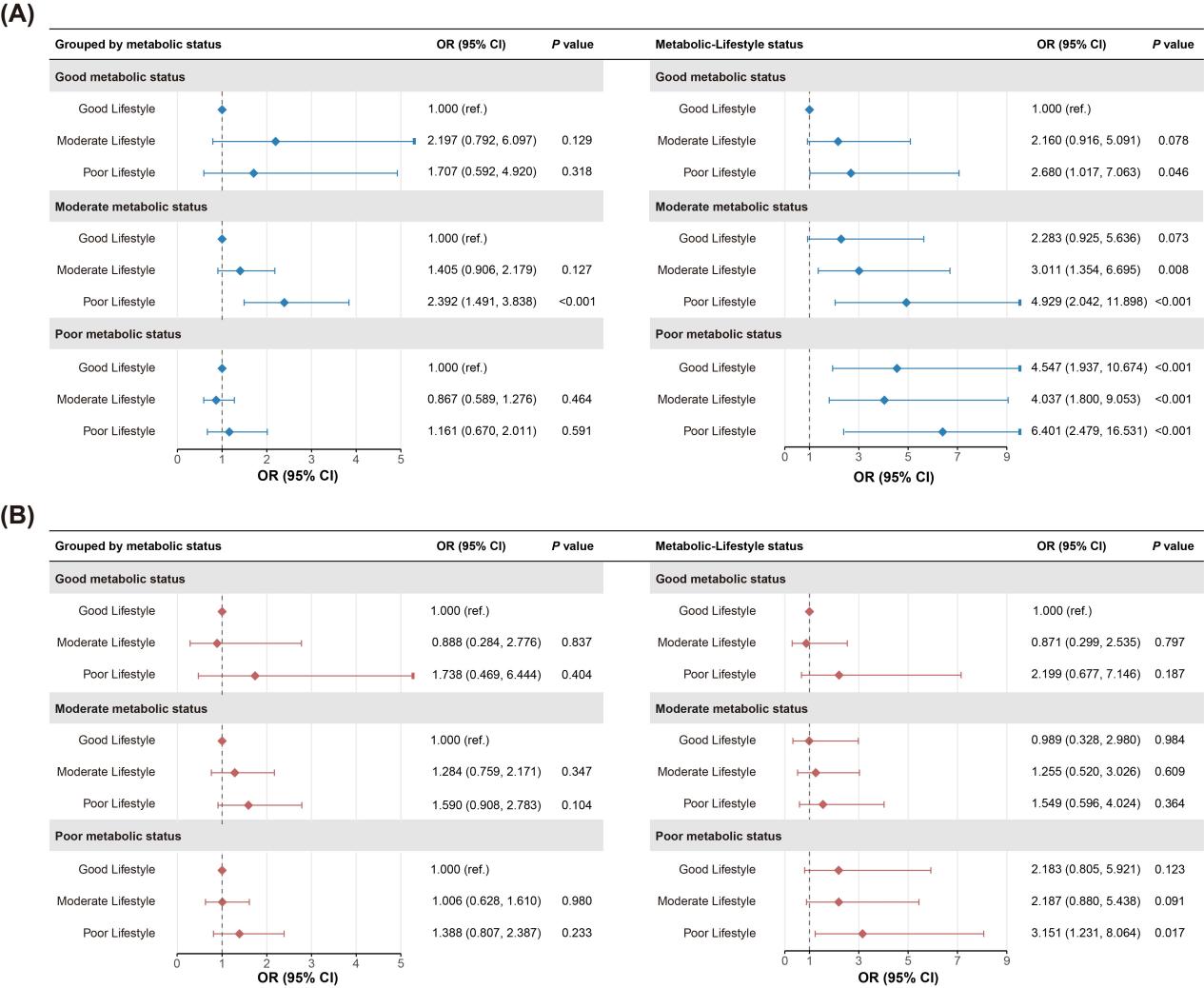


**Supplementary Figure S10** Combined associations of metabolic health and lifestyle with back- or neck-related functional limitation: sensitivity analyses by sedentary behavior. (A) non-sedentary populations; (B) sedentary populations.

Left panels present associations between lifestyle status and outcomes within each metabolic status category. Right panels present joint analyses across nine combined metabolic-lifestyle categories, using participants with both good metabolic and lifestyle status as the reference group.

Models were adjusted for age, sex, race/ethnicity, income, education, marital status, and history of cancer/malignancy.


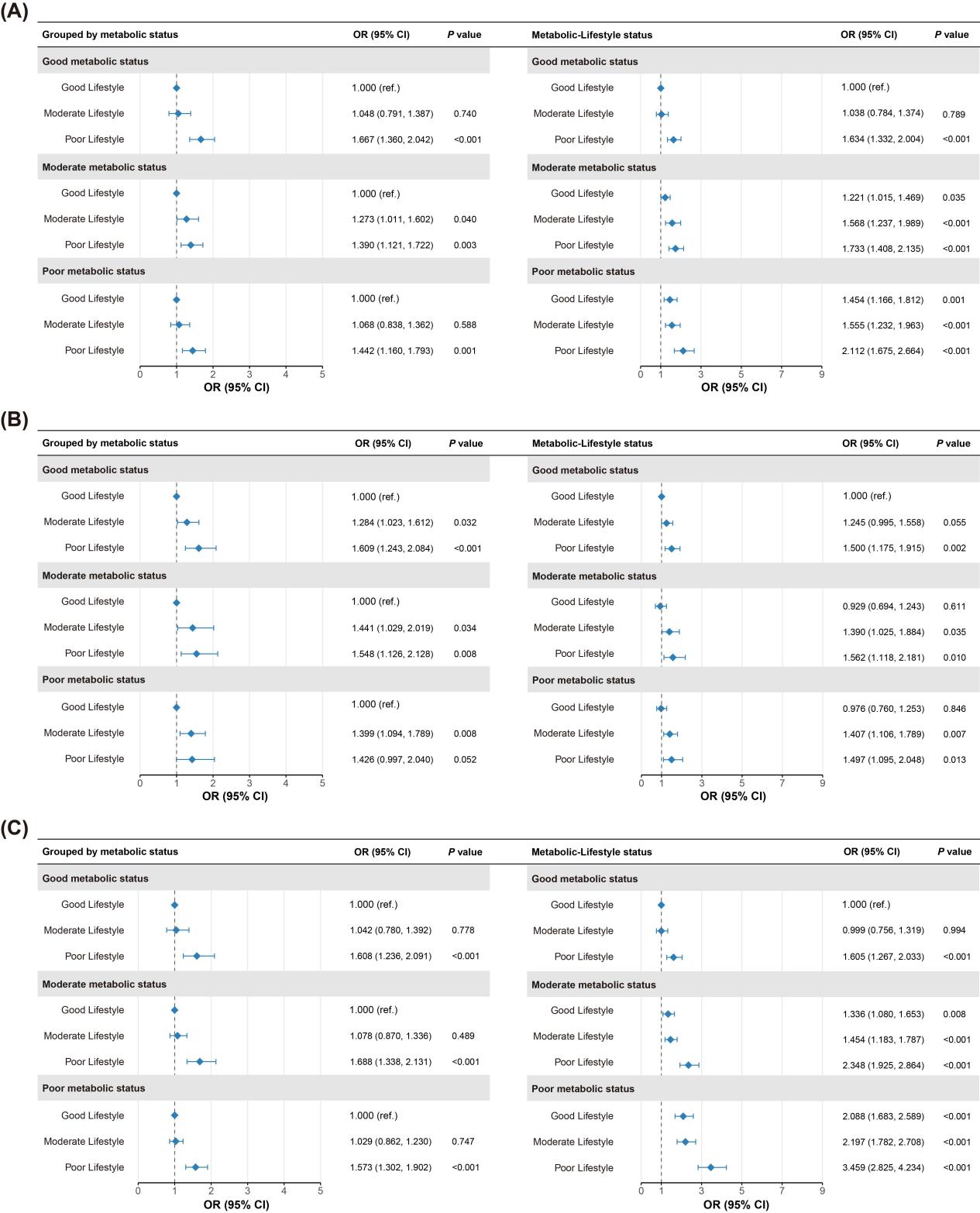


**Supplementary Figure S11** Combined associations of metabolic health and lifestyle with outcomes: sensitivity analyses by weighted scoring. (A) low back pain; (B) neck pain; (C) back- or neck-related functional limitation.

Weighted metabolic and lifestyle scores were constructed based on regression coefficients. Each risk factor was coded as a binary variable (“yes” = 1, “no” = 0) and multiplied by its corresponding weight to calculate the final score. Participants were then classified into three categories based on tertiles of the score distribution: good (Q1), moderate (Q2), and poor (Q3). Models were adjusted for age, sex, race/ethnicity, income, education, marital status, and history of cancer/malignancy.
